# The Brain Basis for Misophonia

**DOI:** 10.1016/j.cub.2016.12.048

**Published:** 2017-02-20

**Authors:** Sukhbinder Kumar, Olana Tansley-Hancock, William Sedley, Joel S. Winston, Martina F. Callaghan, Micah Allen, Thomas E. Cope, Phillip E. Gander, Doris-Eva Bamiou, Timothy D. Griffiths

**Affiliations:** 1 Institute of Neuroscience, Medical School, Newcastle University, Newcastle upon Tyne NE2 4HH, UK; 2 Wellcome Trust Centre for Neuroimaging, 12 Queen Square, London WC1N 3BG, UK; 3 Institute of Cognitive Neuroscience, 17 Queen Square, London WC1N 3AR, UK; 4 Department of Clinical Neurosciences, University of Cambridge, Cambridge CB2 0SZ, UK; 5 Human Brain Research Laboratory, Department of Neurosurgery, The University of Iowa, Iowa City, IA 52242, USA; 6 UCL Ear Institute, 332 Grays Inn Road, London WC1X 8EE, UK

**Keywords:** misophonia, affective disorders, fMRI, functional connectivity, autonomic response, interoception

## Abstract

Misophonia is an affective sound-processing disorder characterized by the experience of strong negative emotions (anger and anxiety) in response to everyday sounds, such as those generated by other people eating, drinking, chewing, and breathing [1, 2, 3, 4, 5, 6, 7, 8]. The commonplace nature of these sounds (often referred to as “trigger sounds”) makes misophonia a devastating disorder for sufferers and their families, and yet nothing is known about the underlying mechanism. Using functional and structural MRI coupled with physiological measurements, we demonstrate that misophonic subjects show specific trigger-sound-related responses in brain and body. Specifically, fMRI showed that in misophonic subjects, trigger sounds elicit greatly exaggerated blood-oxygen-level-dependent (BOLD) responses in the anterior insular cortex (AIC), a core hub of the “salience network” that is critical for perception of interoceptive signals and emotion processing. Trigger sounds in misophonics were associated with abnormal functional connectivity between AIC and a network of regions responsible for the processing and regulation of emotions, including ventromedial prefrontal cortex (vmPFC), posteromedial cortex (PMC), hippocampus, and amygdala. Trigger sounds elicited heightened heart rate (HR) and galvanic skin response (GSR) in misophonic subjects, which were mediated by AIC activity. Questionnaire analysis showed that misophonic subjects perceived their bodies differently: they scored higher on interoceptive sensibility than controls, consistent with abnormal functioning of AIC. Finally, brain structural measurements implied greater myelination within vmPFC in misophonic individuals. Overall, our results show that misophonia is a disorder in which abnormal salience is attributed to particular sounds based on the abnormal activation and functional connectivity of AIC.

## Highlights

• Trigger sounds elicit exaggerated response in anterior insula in misophonia
• In misophonia, there is abnormal functional connectivity of anterior insula
• Heightened autonomic responses are mediated by anterior insula in misophonia
• Misophonia is associated with altered interoception

## Teaser

Kumar et al. show that misophonia is associated with abnormal activation, functional connectivity, and structural changes in the brain and heightened autonomic responses of the body.

## Results and Discussion

**Figure 1:**
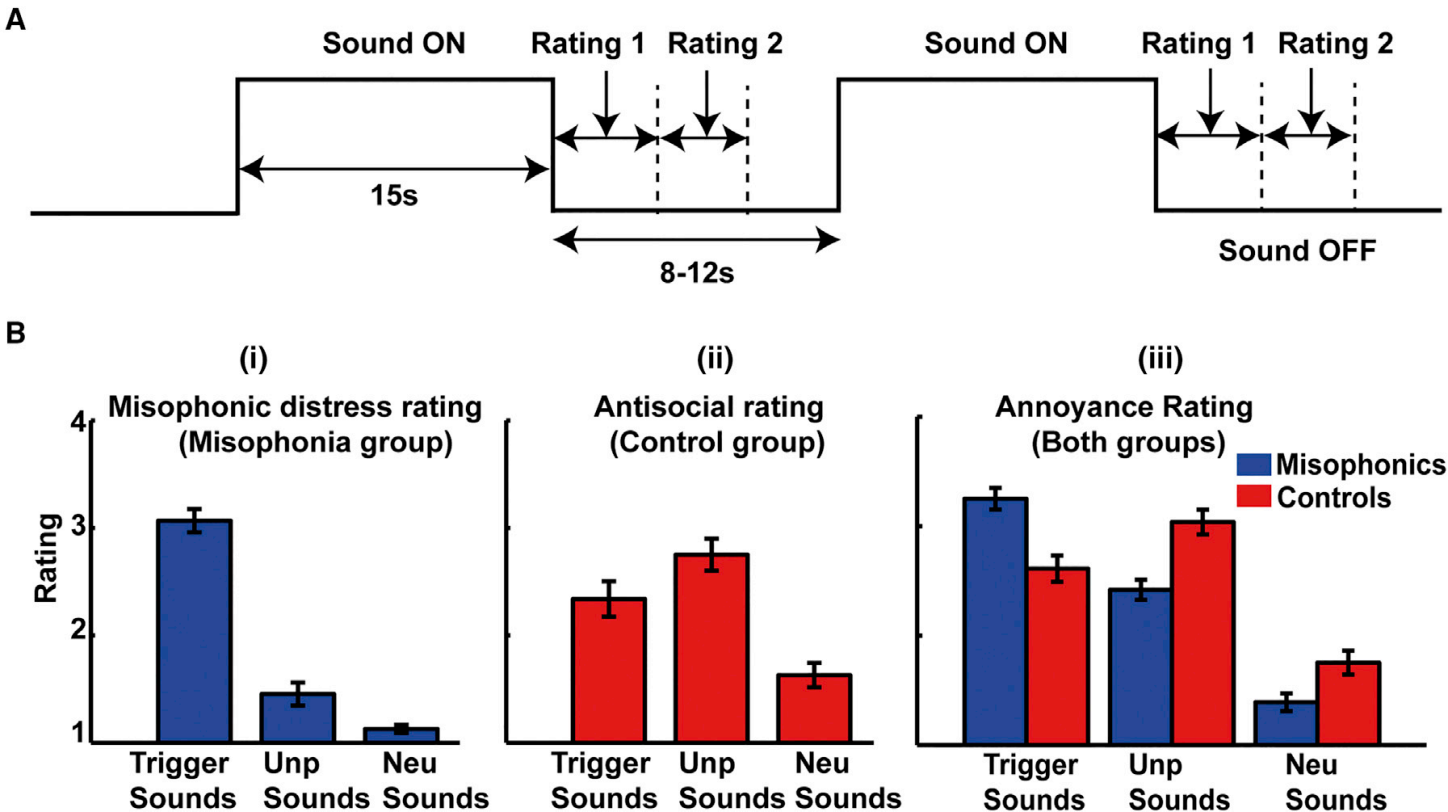
Experimental Paradigm and Subjective Ratings (A) fMRI paradigm: a standard block design was used in which sounds were presented for 15 s. After every sound, subjects gave two ratings on a scale from 1 to 4 with a button press for (1) how annoying the sound was and (2) how effective the sound was in triggering misophonic reaction (misophonia group) or how antisocial the sound was (control group). fMRI data were acquired continuously with a repetition time (TR) of 3.12 s. GSR and HR were also monitored throughout the experiment. (B) Subjective ratings: (i) misophonic distress rating of three types of sounds by misophonic group; (ii) antisocialness rating of sounds (control subjects); and (iii) annoyance rating of sounds by both groups. Misophonic subjects rated the trigger sounds as evoking greater misophonic reaction compared to unpleasant (p < 0.001) and neutral sounds (p < 0.001). Unpleasant sounds were still perceived to be annoying (p < 0.001 compared to neutral sounds) by the misophonic subjects, demonstrating a dissociation between general annoyance and misophonic reaction. See also Figure S4 for subjective scores on body perception. Data are represented as mean (±SEM).

fMRI data were acquired in 20 misophonic and 22 age- and sex-matched controls while they listened to a set of three sounds: trigger sounds (which evoke a misophonic reaction in misophonic individuals; e.g., eating, breathing sounds), unpleasant sounds (which are perceived to be annoying by both groups but do not evoke misophonic distress; e.g., baby cry, a person screaming), and neutral sounds (e.g., rain). After listening to each sound, subjects rated (1) how annoying the sound was (both groups) and (2) how effectively the sound triggered a typical misophonic reaction (misophonic group only) or how antisocial (in the sense the subject would not like to be in the environment in which the sound is produced) the sounds were (control group only). Behavioral responses, galvanic skin response (GSR) and heart rate (HR), were acquired during the acquisition of fMRI data (see Figure 1A for a schematic of the paradigm). Whole-brain structural MRI data were acquired as multi-parameter maps (MPMs) [9] to measure myelination content, water, and iron levels.

**Figure 2:**
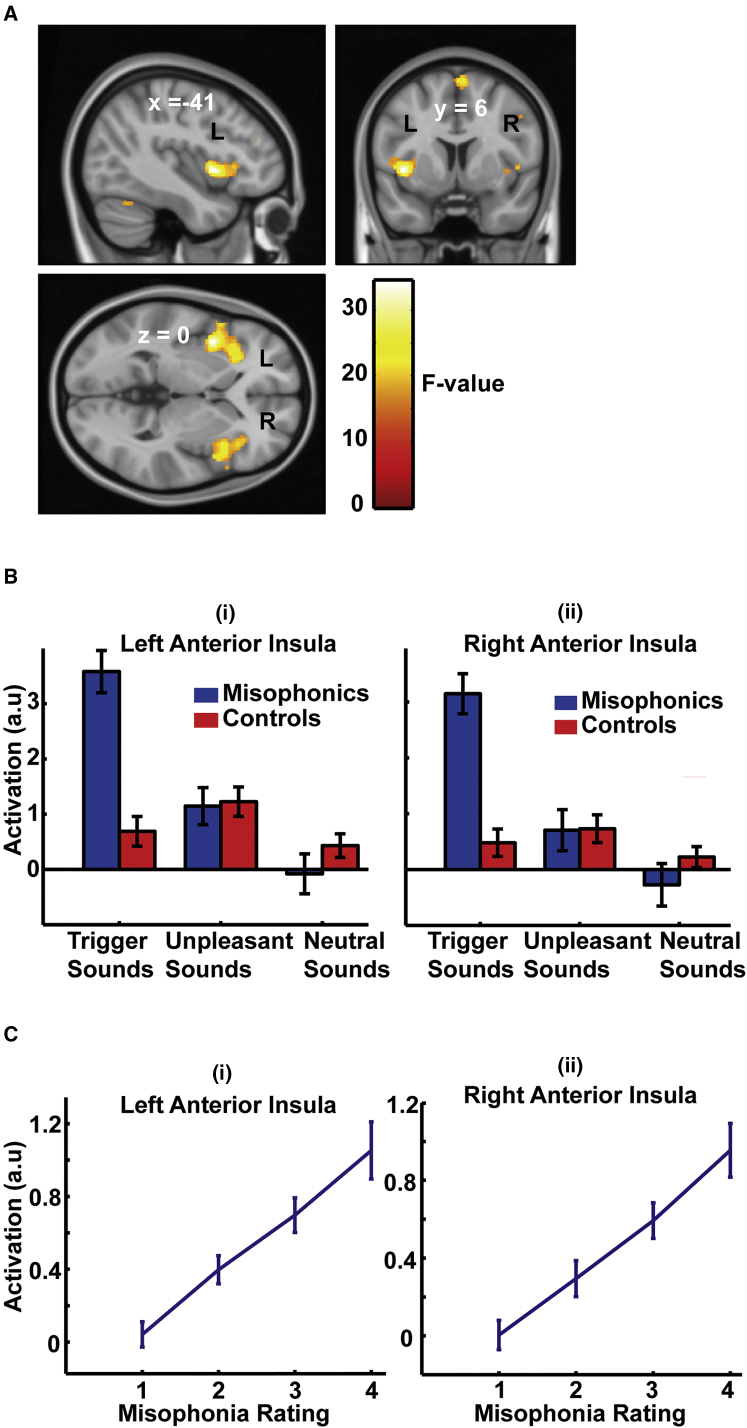
Group-Level, Random-Effects GLM Analysis of fMRI Data The GLM was modeled as a factorial design with group (two levels) and sound types (three levels) as factors. (A) Statistical parameter maps (SPMs) overlaid on a standard MNI-152 template brain for the critical interaction between the two factors (group and sound type) thresholded at p = 0.05 family-wise error (FWE) corrected for whole-brain volume. The effect is maximal in AIC (bilateral) with maxima at MNI coordinates (−41, 6, 0). (B) Confirmatory plots of activity averaged over cluster in AIC (see also Figures S1 and S2 and Table S1) show that the interaction effect was driven by higher activity for trigger sounds in misophonic subjects compared to controls. (C) Confirmatory plots of activity in AIC with misophonic ratings in misophonic subjects. Data in (B) and (C) show mean (± SEM).

Behavioral data (Figure 1B) showed that trigger sounds evoked misophonic distress in misophonic subjects, whereas the unpleasant sounds, although annoying, did not produce a misophonic reaction. There was no difference between the misophonic distress ratings of trigger sounds by the misophonic group and annoyance ratings of unpleasant sounds by the control group. It is likely, however, that the two groups used different subjective scales while rating the sounds. Random-effects analysis of fMRI data using the general linear model (GLM) [10] with group (two levels) and sound types (three categories) as factors demonstrated an interaction in the anterior insular cortex (AIC) bilaterally (Figure 2A; further regions are listed in Table S1). Further analysis showed that the interaction in AIC was driven by greater activation in misophonic subjects compared to control subjects in response to trigger sounds (see Figure 2B and Figure S1 for confirmatory plots; see also Figure S2). Significant activation differences between misophonic and control subjects did not occur to unpleasant or neutral sounds. Activity in both the left and right AIC varied linearly with the subjective rating of misophonic distress in the misophonic group, as shown in confirmatory plots in Figure 2C. A large body of evidence [11] implicates AIC in subjective feelings associated with emotions, including anger. Functionally, AIC is known to be a key node of the salience network [12], an intrinsic large-scale brain network for detecting and orienting attention toward stimuli that are behaviorally relevant and meaningful for an individual. Specific hyperactivity in AIC to trigger sounds supports the hypothesis that misophonic subjects assign aberrantly higher salience to these sounds.

**Figure 3:**
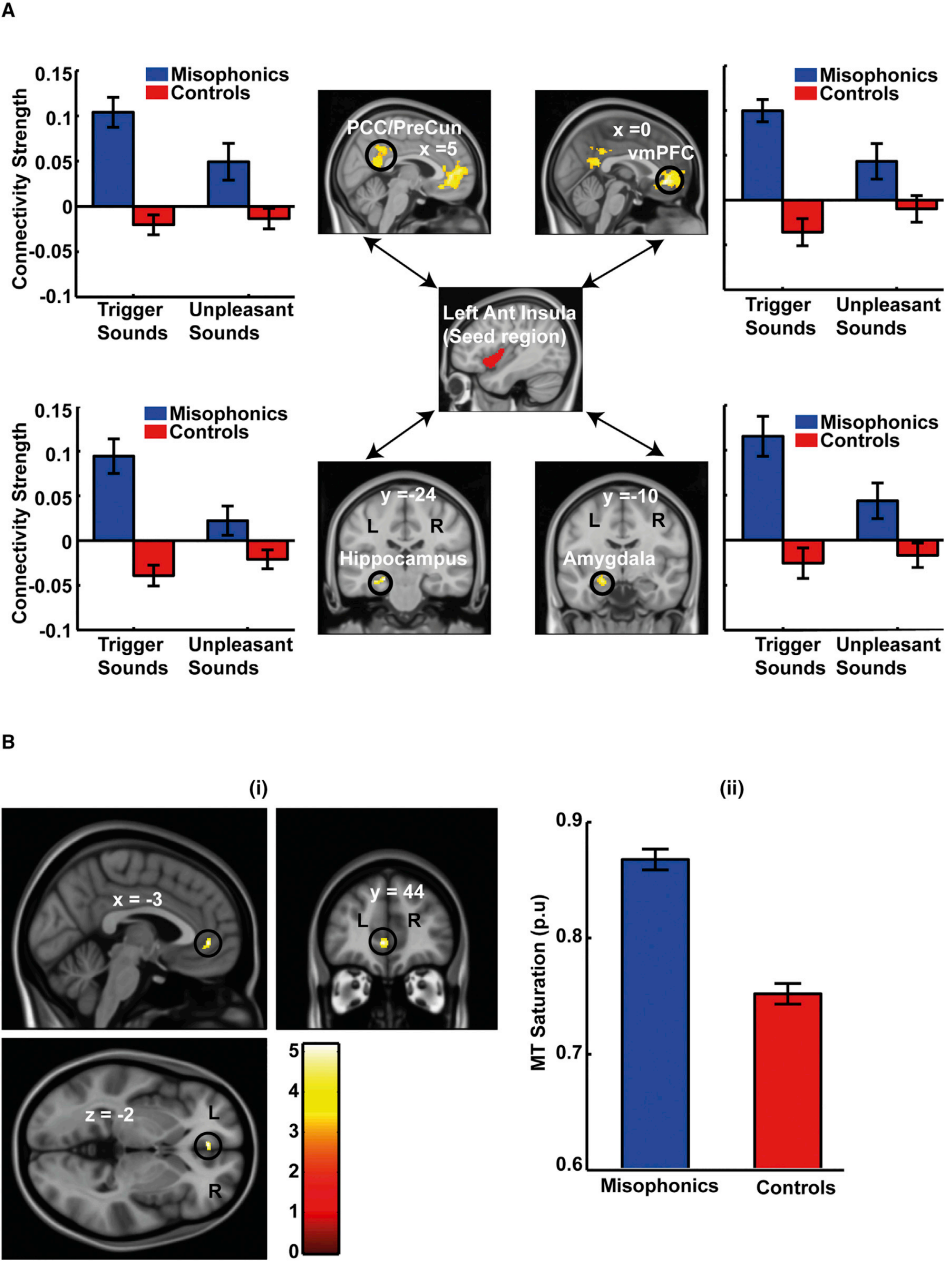
Functional Connectivity and Structural Data Analysis (A) Left AIC was taken as a seed region and its functional connectivity to all voxels of the brain was analyzed. The figure illustrates those brain areas that show greater connectivity for trigger sounds (compared to neutral sounds) in misophonic subjects (compared to controls). The four areas that survive the threshold are (1) PMC (posterior cingulate cortex [PCC]/precuneus), (2) vmPFC, (3) hippocampus, and (4) amygdala. The bar chart for each region shows confirmatory plots of connectivity for trigger and unpleasant sounds with respect to neutral sounds. Displayed connectivity strengths are cluster thresholded at p < 0.05 with cluster-forming threshold at p < 0.001 (see Figure S3 for functional connectivity of right AIC and overlap of the connectivity network with the default mode network). (B) Brain structural changes in misophonia. Misophonic subjects show higher MT saturation, which reflects higher myelination, compared to controls in vmPFC. When corrected for multiple comparisons (p < 0.05 FWE corrected for brain areas that show higher functional connectivity in misophonics to trigger sounds; i.e., the functional network shown in (A) along with the seed region AIC), 15 voxels of vmPFC with maxima at (−3, 44, −2) survive the correction. For display purposes in the figure, a threshold of p < 0.001 uncorrected is used. p.u., percent units. Data in bar charts show mean (± SEM).

Having identified AIC as a key region that differentiates trigger sounds in misophonic participants, we sought to explore its stimulus-dependent connectivity profile to establish whether there are alterations at the network level that are specific to misophonia. Using left AIC as a seed region, we analyzed its stimulus-dependent connectivity in the two groups. Greater functional connectivity of AIC for misophonic subjects was observed in a network of brain regions comprising the ventromedial prefrontal cortex (vmPFC), posteromedial cortex (PMC; posterior cingulate and retrosplenial cortex), hippocampus, and amygdala (Figure 3A). This increased functional connectivity was specific to trigger sounds: no significant differences in connectivity were observed for unpleasant sounds. Importantly, the functional connectivity pattern between the two groups for the same sounds was not only different quantitatively but also qualitatively: whereas the connectivity to vmPFC is positive (with respect to connectivity for neutral sounds) in misophonic subjects, the connectivity for controls for the same set of sounds is negative. Analysis of functional connectivity of right AIC also showed trigger-sound-specific increased connectivity to vmPFC and PMC (Figure S3A; functional connectivity to amygdala and hippocampus was also observed but at a slightly relaxed threshold). The vmPFC and PMC together form core parts of the default mode network (DMN) [13] (see Figure S3B for overlap between the DMN and the functional connectivity network of AIC), which is activated when subjects are engaged in internally directed thoughts and retrieval of memories [14] and is deactivated when attention is directed to external stimuli. Greater coupling of AIC with the DMN suggests that misophonic subjects, on hearing trigger sounds, are unable to “disengage” AIC from the DMN, which entails memories and contextual associations of trigger sounds to bear on the activation of AIC. This is also consistent with a recent study [15] using multivariate pattern classification, which showed that patterns of activity in vmPFC and PMC were most informative in distinguishing different types of emotions. Distinct functional connectivity of AIC to vmPFC and PMC in misophonics and controls for the same sounds suggests that these regions play a crucial role in instantiating different emotional responses for the trigger sounds in the two groups. This atypical functional connectivity could, therefore, underlie the abnormal activation of AIC and the aberrant salience assigned to trigger sounds by the misophonic group.

Because misophonia symptoms start early in life (mean age of onset is ∼12 years and can be as early as 5 years [1]), we also predicted that there would be brain structural differences in misophonic subjects compared to controls. We created whole-brain structural maps of magnetization transfer (MT) saturation that reflects myelination in brain gray matter. For significance testing, we limited our search to brain areas that showed higher functional connectivity to AIC in misophonics compared to controls along with the seed region. Analysis of structural maps showed that misophonic subjects have altered MT saturation, which is consistent with significantly higher myelination in the gray matter of vmPFC (Figure 3B). This change suggests a possible structural basis for the altered functional connectivity to vmPFC observed in misophonic subjects.

**Figure 4:**
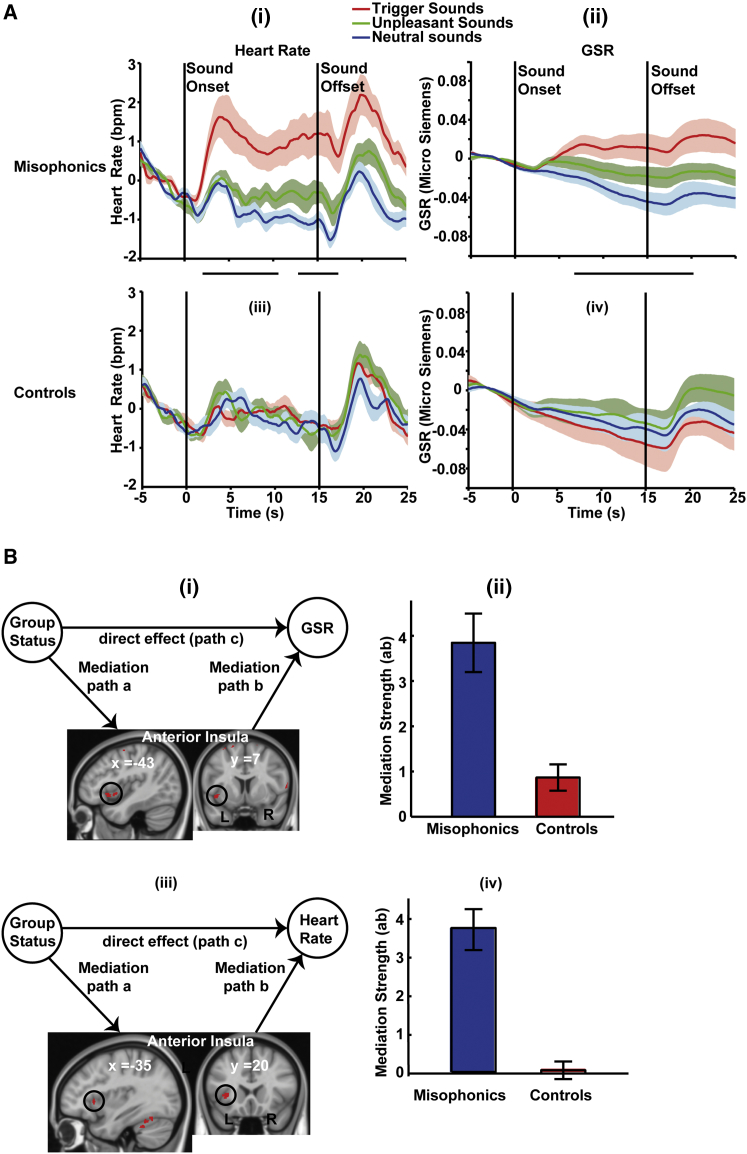
Psychophysiological Responses and Their Mediation by Brain Areas (A) HR and GSR for misophonic and control subjects. In misophonic subjects, the trigger sounds produce sustained increases in HR and GSR. Statistical analysis of GSR and HR was performed time by time using a 2 × 3 ANOVA as in the fMRI analysis. For the HR time series, interaction between the factors was significant from 2.4 to 10.4 s and then from 12.4 to 17 s after sound onset. For the GSR time series, significant interaction was observed from 7 to 21.4 s after sound onset (time points at which GSR and HR are significantly different are indicated by black horizontal bars between the panels). Both HR and GSR time series were cluster thresholded at p < 0.05 with cluster-forming threshold at p < 0.05. Post hoc comparison showed that the interaction effect in both HR and GSR was driven by higher responses to trigger sounds in misophonic subjects. There was no difference between the two groups in their responses to unpleasant and neutral sounds. bpm, beats per min. (B) Mediation analysis to determine which brain areas mediate the increased HR and GSR in misophonic subjects, relative to controls, to trigger sounds. Whole-brain, single-level mediation analysis was used, in which the input X is a categorical vector (+1 for misophonics and −1 for controls) and the response vector Y contains an average increase in HR/GSR (compared to neutral sounds) over a trial of trigger sounds for each subject. The mediation variable M is the beta value (as determined using SPM) for trigger sounds compared to neutral sounds. (i) Left AIC mediates GSR changes. (ii) Confirmatory plots of mediation strength for GSR for the two groups averaged over the cluster in AIC. (iii) AIC mediates heightened HR in misophonics. (iv) Confirmatory plots of mediation strength for HR for the two groups averaged over the cluster in AIC. The displayed results (i) and (iii) are thresholded at p < 0.005 with a cluster extent threshold of 50 voxels. Data are represented as mean (± SEM; shaded areas in A and error bars in B).

After identification of functional and structural changes in the brain, we next determined physiological responses of the body and their driving sources in the brain. We measured GSR and HR while subjects listened to three sets of sounds in the MRI scanner. Trigger sounds evoked greater GSR and HR responses in misophonic subjects than control subjects (Figure 4A). Physiological responses were sustained throughout the duration of sound presentation and were specific to trigger sounds, with no difference in GSR or HR response between the two groups for unpleasant and neutral sounds. The heightened trigger-specific autonomic responses we observed are consistent with the strong tendency of misophonic subjects to escape from the environment of trigger sounds [1, 2] or experience strong anxiety and anger if unable to escape (fight/flight response).

What is the brain source(s) of these heightened autonomic responses in misophonia? To answer this, we used mediation analysis [16], which aims to test whether a relation from variable X (group membership; i.e., misophonic or control) to Y (GSR or HR) could be explained (mediated) by a third variable, M (brain activation). A significant mediation implies that there is an indirect path to Y (X to M to Y) and would show brain activity (M) that can mediate the observed GSR/HR (Y) over and above what is explained by group membership (X). We ran the whole-brain mediation analysis separately for GSR and HR. We found that activity in AIC mediated both the heightened GSR and HR (Figure 4B) in misophonic subjects.

Over the last decade, there has been a growing recognition that interoception (perception of internal bodily states) can influence the salience and experience of emotions associated with a stimulus [17, 18, 19, 20]. Interestingly, AIC is the key brain structure that integrates ascending visceral inputs from the body with external sensory inputs. In accordance with this, atypical interoception and activation in AIC have been shown to underlie a number of social-emotional disorders [21, 22]. Recently, there has been a growing interest in extending prediction-based hierarchical Bayesian inference as a model of interoception [19, 23]. In this model, interoception involves inferring causes of interoceptive signals by combining bottom-up interoceptive signals with prior beliefs (predictions) of their causes. In this multi-level and hierarchically organized inference scheme, AIC is at the top of the hierarchy and is suggested to infer the overall state of the body [24]. Evaluation of subjective beliefs about body perception using the Body Consciousness Questionnaire [25] showed that misophonics report greater awareness of internal sensations (Figure S4) compatible with altered interoceptive sensibility [22] in misophonics. Given the role of AIC in representing bodily states, the questionnaire data are also consistent with abnormal AIC functioning in misophonia.

### Conclusions

Overall, our data show that for misophonics, trigger sounds cause hyperactivity of AIC and an abnormal functional connectivity of this region with medial frontal, medial parietal, and temporal regions; that there is abnormal myelination in medial frontal cortex that shows abnormal functional connectivity to AIC; and that the aberrant neural response mediates the emotional coloring and physiological arousal that accompany misophonic experiences. Together, our data suggest that abnormal salience attributed to otherwise innocuous sounds, coupled with atypical perception of internal body states, underlies misophonia. With the available data, it is not possible to decide whether misophonia is a cause or consequence of atypical interoception, and further work is needed to delineate the relation between the two.

Misophonia does not feature in any neurological or psychiatric classification of disorders; sufferers do not report it for fear of the stigma that this might cause, and clinicians are commonly unaware of the disorder. This study defines a clear phenotype based on changes in behavior, autonomic responses, and brain activity and structure that will guide ongoing efforts to classify and treat this pernicious disorder.

## Author Contributions

S.K., O.T.-H., W.S., J.S.W., and T.D.G. designed the experiments; S.K. and O.T.-H. collected the data; S.K., with help from W.S., J.S.W., M.F.C., and T.D.G., analyzed the data; S.K., O.T.-H., W.S., J.S.W., M.F.C., M.A., T.E.C., P.E.G., D.-E.B., and T.D.G. wrote the paper; and T.D.G. supervised all aspects of the work.

## Supplemental Information

Document S1. Supplemental Experimental Procedures, Figures S1–S4, Table S1, and Questionnaire

Document S2. Article plus Supplemental Information
