# Supplementary material for: The Brain Basis for Misophonia
Source: Curr Biol. 2017 Feb 20;27(4):527–33. doi: 10.1016/j.cub.2016.12.048 (PMC5321671; doi:10.1016/j.cub.2016.12.048)
Supplement: Document S1. Supplemental Experimental Procedures, Figures S1–S4, Table S1, and Questionnaire [file mmc1.pdf]

Current Biology, Volume *27*

Supplemental Information

The Brain Basis for Misophonia

Sukhbinder Kumar, Olana Tansley-Hancock, William Sedley, Joel S. Winston, Martina F. Callaghan, Micah Allen, Thomas E. Cope, Phillip E. Gander, Doris-Eva Bamiou, and Timothy D. Griffiths

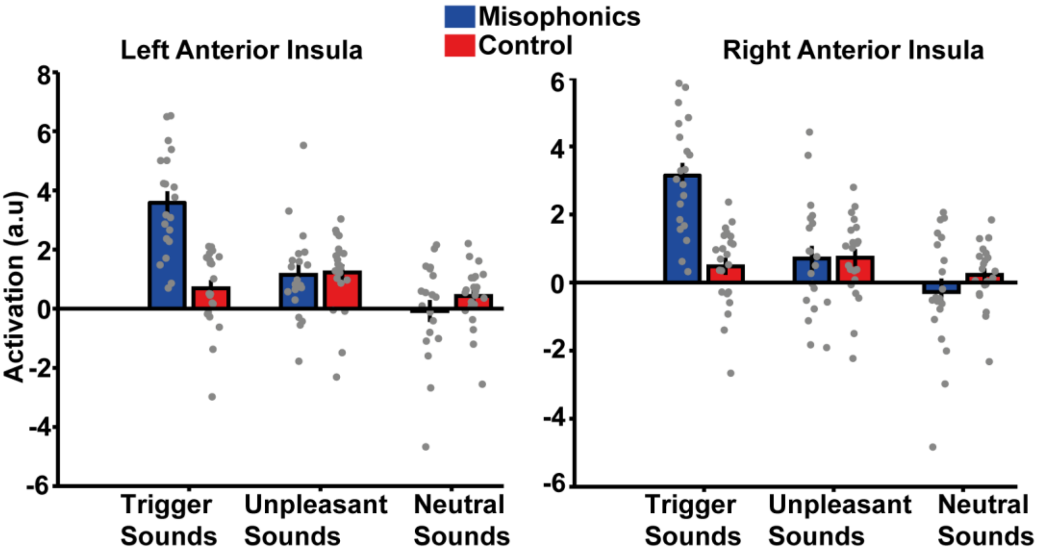

Figure S1: **Mean and distribution of anterior insula response; Related to Figure 2**. Plots of individual subject data along with the mean and standard error for activation (as determined by general linear model analysis) in the left and right anterior insula for both groups. Data in the bars are represented as mean (± SEM).

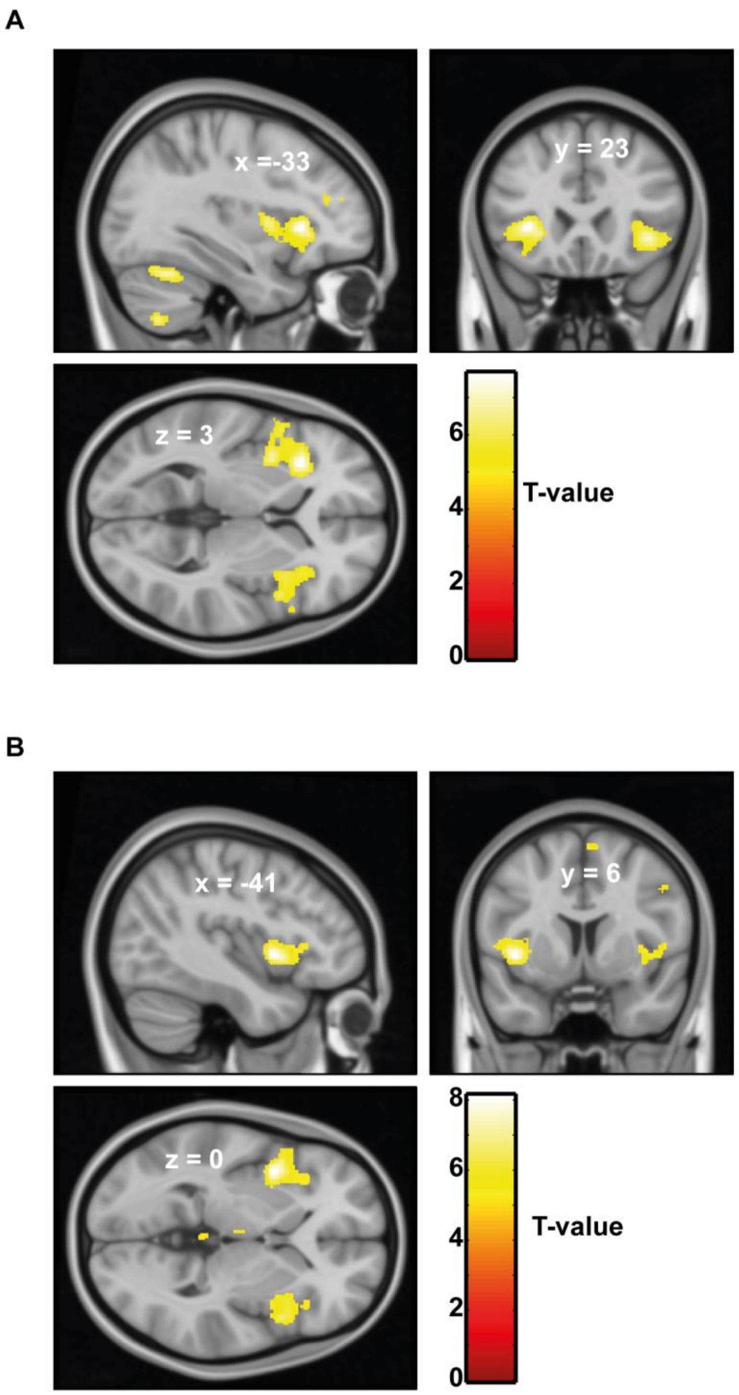

Figure S2: Post-hoc testing for the interaction in GLM analysis; Related to Figure 2. (A) Statistical parametric map showing brain regions with greater BOLD activation in misophonics compared to controls in response to trigger sounds compared to neutral sounds. Specifically the contrast as defined in SPM is: Misophonics (Trigger sounds- Neutral sounds) > Controls (Trigger sounds- Neutral sounds). **(B)** Activation of trigger sounds compared to unpleasant sounds: parametric map for the contrast Misophonics (Trigger sounds- Unpleasant sounds) > Controls (Trigger sounds- Unpleasant sounds). The parametric maps in both panels are thresholded at p =0.05 (FWE corrected for the whole brain volume).

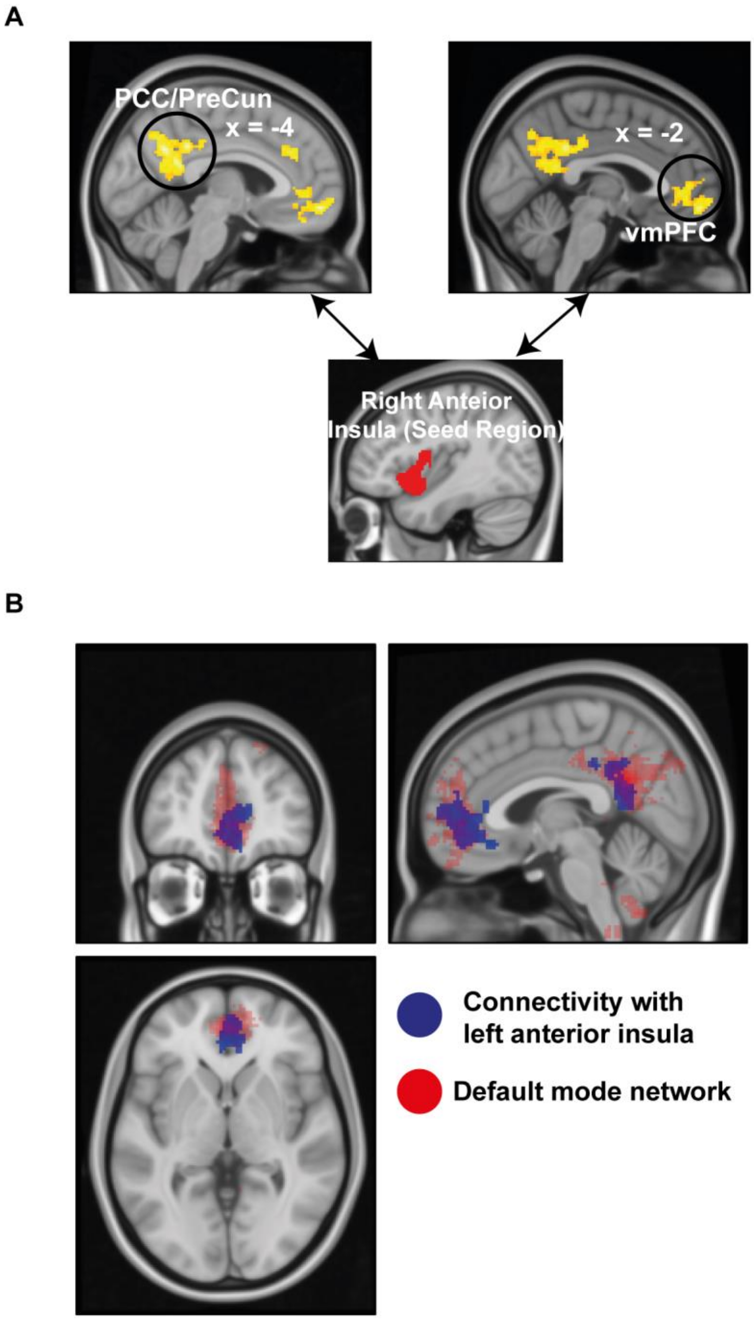

Figure S3: **Functional connectivity of anterior insula and its overlap with default mode network; Related to Figure 3** (A) Functional connectivity with right AIC as a seed region. The figure shows increased connectivity of right AIC to vmPFC and PMC for trigger sounds. The defined contrast is: Misophonics (Trigger sounds- Neutral sounds) > Controls (Trigger sounds- Neutral sounds). The connectivity map is cluster thresholded at $p < 0.05$ with cluster-forming threshold at $p < 0.001$. Other areas which show increased connectivity are, left inferior parietal (-40, 68, 34), anterior cingulate (-6, 34, 28) and parahippocampal cortex (-20, -36, -18). Hippocampus and amygdala are also seen but when the cluster forming threshold is relaxed to 0.005. **(B)** Overlap between the default mode network (DMN) and functional connectivity of left anterior insula shown in Figure 3. The DMN map was obtained using reverse inference from the Neurosynth website (http://neurosynth.org/analyses/terms/default%20mode/).

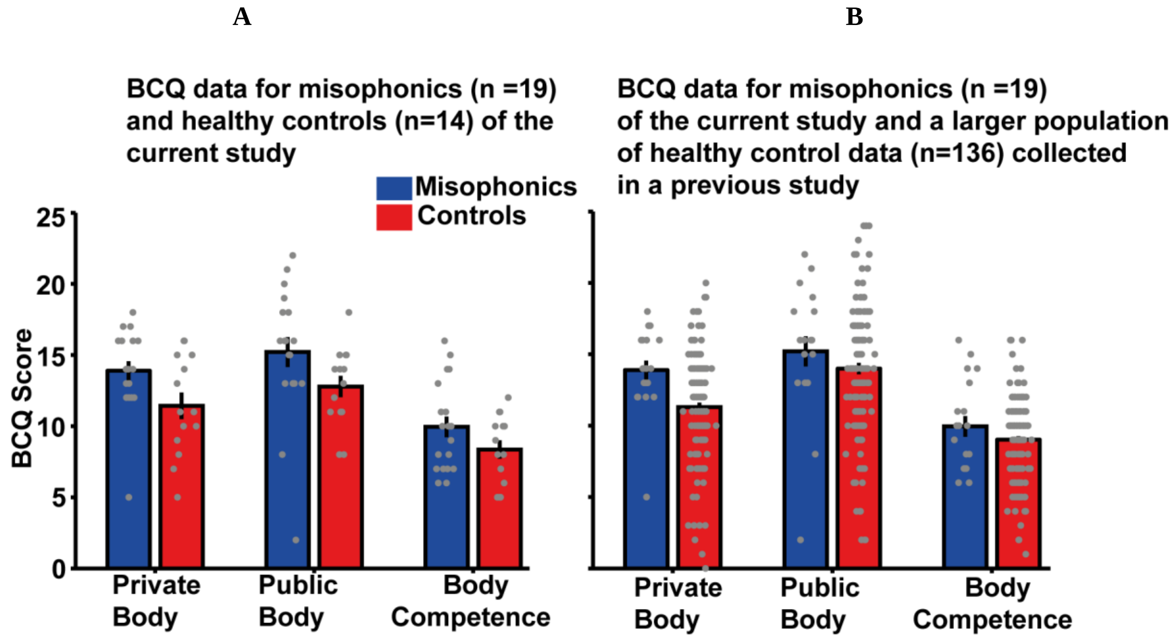

Figure S4: Scores on the three subscales of the body consciousness questionnaire (BCQ); Related to Figure 1. (A) Data from misophonics (n=19) and controls (n=14) who took part in the current study. Statistical comparison (Mann-Whitney U test) between the two groups showed that misophonics scored higher both on the 'private' (p=0.02) and 'public' (p=0.03) categories but were not different from controls on 'body competence' (p= 0.22). **(B)** Comparison of the misophonics scores with data collected from a larger sample (n=136) of normal healthy controls. Misophonics scored higher (p =0.002) on 'private' but were not different from controls in rest of the two categories: 'public' (p = 0.17), 'competence' (p= 0.42). In order to rule out effect of age and gender in the above analysis, we also compared the two groups by tightly matching each misophonic in terms of age (±3 years) and gender of control participants (n=2-132) and looking for systematic effects with the sign test. This comparison showed misophonics scored more highly (p = 0.02) on 'private' but were not different on 'public' (p= 0.65) and 'body competence' (p = 0.36). Dots in the figure show score of individual subjects. For details of the questionnaire, see the section on 'Questionnaire data analysis in supplemental experimental procedure. Data are represented as mean (± SEM).

Table S1: **Brain areas which show interaction between group and sound type. The SPM is thresholded at p < 0.05 (FWE corrected for the whole brain volume). Related to Figure 2.**

Brain Area name	MNI coordinates	F-value at the maxima	Size of the cluster
Left anterior insula	-41, 6, 0	34.37	1720
Left dorsal anterior insula	-33, 23, 3	30.18	
Left inferior frontal gyrus	-51, 2, 11	27.42	
Left cerebellum	-30, -65, -53	29.20	207
	-27, -65, -24	29.07	522
	-36, -57, -26	26.55	
	-45, -63, -29	16.56	
Right inferior frontal gyrus	48, 11, 2	26.87	1140
Right anterior insula	39, 23, -3	25.18	
Right anterior insula	35, 30,0	19.72	
Right SMA	5, 8, 66	23.90	289
	5, -3, 68	20.35	
Right cerebellum	32, -63, -23	22.47	223
	35, -56, -27	18.91	
	21, -68, -47	19.06	16
	32, -56, -50	18.97	55
	27, -65, -54	16.83	6
Left middle frontal gyrus	-38, 39, 21	20.30	42
Right brain stem	9, -30, -11	19.31	10
	9, -41, -44	16.91	2
	5, -45, -47	16.64	1
Left supramarginal gyrus	-59, -41, 29	18.05	12
Left thalamus	-11, -23, -11	17.49	8
Right precentral gyrus	48, 5, 39	17.19	18
	47, 2, 53	16.68	5
Left cingulate sulcus	-3, 27, 33	16.95	8
Left superior frontal gyrus	-50, 12, 29	16.09	1

Supplemental Experimental Procedures

Subjects

Twenty four misophonic subjects (16 females, mean age 33.8 years, age-range: 18-57 years) and 22 control subjects (15 females, mean age-range 32.5 years, age-range: 19-57 years) participated in the study after providing written informed consent to procedures approved by the local ethics committee. The misophonic subjects were recruited by putting an advertisement on a misophonia support website: http://www.misophonia-uk.org/. Misophonic participants were first required to complete a questionnaire (Please see the supplemental Questionnaire S1). A misophonic participant was recruited for the study if (i) he/she identified sounds of eating, breathing, chewing as trigger sounds (ii) sounds alone could trigger the misophonic reaction (that is, no picture or video of the person producing trigger sounds was needed along with sounds) (iii) the person producing trigger sounds did not have to be a close family member (that is, a stranger producing trigger sounds could produce similar if not the same reaction). The controls were recruited by advertising on a local university website. In the advertisement, the exact purpose of the study was not mentioned. Instead it was stated that the objective of the study was to determine brain responses to our day-to-day environmental sounds. Once participants signed up for study, they were asked how they respond to environmental sounds including sounds of eating, breathing. If typical symptoms of misophonia were absent (e.g. responding angrily, leaving the place) the subject was recruited. No subject who signed up for the study was incidentally diagnosed as misophonic. The misophonics and controls were matched in age and sex. Four misophonic subjects could not be included in the analysis because one subject did not complete the full paradigm (because of emotional distress) and three subjects moved excessively in the MRI scanner. Participants were paid £10/hour plus travel expenses, if any, for their participation.

Stimuli

One objective of the study was to test if misophonic reactions were related to the typical annoyance that is experienced by most people when listening to certain sounds such as sound of a person screaming or a baby cry. Our experiment, therefore included a set of unpleasant sounds (expected to prove annoying to both misophonic and control group) in addition to a set of common misophonic trigger sounds which evoke a misophonic reaction in susceptible individuals. Additionally a set of affectively neutral sounds were used as control sounds. In summary, the experimental stimuli consisted of three sets of sounds (1. Trigger sounds, e.g. eating, breathing, drinking sounds) 2. Unpleasant sounds, e.g. baby cry, a person screaming 3. Neutral sounds, e.g. sounds of a busy café, rain sound), each consisting of 14 stimuli. The trigger sounds were recorded in our lab while people were eating, breathing or chewing. The unpleasant and neutral sounds were downloaded from internet websites. Sounds were sampled at a rate of 44.1 kHz and trimmed to 15s duration.

Experimental Procedure

The study was divided in two parts and required participants to visit our lab on two separate days (24-48 hours separation between the two parts). The reason for dividing the study into two parts was that we were not sure how the misophonic subjects would respond to trigger sounds in the confined space of MRI scanner. In the first visit subjects were acquainted with the sounds and the MRI scanning environment and in the second visit fMRI data were acquired while subjects listened to the three sets of sounds.

In the first visit, the full paradigm was explained and after informed consent was obtained subjects were seated in a sound-proof room. Two Ag/AgCl electrodes were placed on the middle and ring fingers of left hand to monitor galvanic skin response (GSR). All three sets of sounds were played binaurally over headphones (SENNHEISSER, HD380 Pro http://en-uk.sennheiser.com/headphones) in a pseudo-randomized order using the following paradigm. The start of a trial was indicated by text instructions appearing on the screen ('Sound to Start Soon'). After a gap of 5-7s (chosen randomly), sound was played for 15s. After sound offset, subjects were prompted to give two sequential ratings on a scale from 1 to 4 with a right-handed button press. In the misophonia group the two ratings were (i) 'how annoying the sound was' ("1: Not annoying" and "4: highly annoying") (ii) 'how effective the sound was in triggering misophonic reaction ("1: Not at all" and "4: Highly effective"). The

procedure for control subjects was same except for the second rating when they rated the ‘ant-socialness’ of the sound in the sense that they would not like to be in the environment in which this sound is made. This task was chosen as it made no sense to ask the non-misophonic participants about misophonic experiences and misophonic subjects have a strong tendency to escape from the environment in which trigger sounds are made. A total of 4 sessions (126 trials) each lasting ~12 minutes were used. After this session, subjects were taken to MRI scanner and a structural scan using Multiparameter maps (MPM) [S1] was acquired which took about ~25 minutes. The GSR data collected in this session is not presented here.

In the second visit, after attaching two Ag/AgCl electrodes on the left middle and ring fingers for monitoring GSR and an MR-compatible pulse oximeter (Nonin Medical; Minnesota, USA) on the distal left index finger, subjects lay in the MRI scanner and EPI data were acquired continuously while all three sets of sounds were played binaurally in a pseudorandom order through MRI compatible headphones (http://www.mr-confon.de/en/products.html) at a comfortable volume of approximately 75dBA. As in the first session outside the scanner, after each sound offset subjects were prompted to give two ratings using an MRI-compatible 4-button response pad. The total scanning time was ~56 minutes (divided into 5 sessions).

Functional imaging data acquisition

All imaging data were collected on Siemens 3 Tesla Tim whole-body MRI scanner (Siemens Healthcare, Erlangen Germany) at the Wellcome Trust Centre for Neuroimaging. Functional MRI data were acquired continuously with a 12 channel coil using a sequence that was optimized for acquisition from amygdala and orbitofrontal cortex [S2]. Subject movement was discouraged by instruction and by use of soft padding around the head within the headcoil. The acquisition parameters used were (TR=3.36s; in-plane resolution=3mm isotropic; TE=30ms; 48 slices (covering the whole brain); matrix size=64x74; echo spacing=0.5ms; orientation=transverse; slice tilt=-30° relative to the AC-PC line). A total of 1005 volumes were acquired across 5 sessions. Fieldmaps were acquired (parameters: short TE=10ms; Long TE=12.46ms; polarity of phase-encoding blips=-1; EPI readout time=37ms) for every subject after third session.

Structural data acquisition

A whole brain quantitative MPM protocol (with 32 channel headcoil) was used which consisted of a total of 5 sequences: three FLASH sequences and two calibration sequences for correcting field inhomogeneities [S3,S4]. The three FLASH sequences were respectively proton density (PD), magnetization transfer (MT) and T1 weighted by choosing appropriate values of repetition time (TR) and flip angle (α) for each of them. The repetition times and flip angles for the three FLASH sequences were (PD: TR=23.7ms, α=6°; MT: TR=23.7ms, α=6°; T1: TR=18.7ms, α=20°). For the MT-weighted acquisition, a Gaussian RF pulse of 4ms duration and 220° nominal flip angle was applied 2 kHz off-resonance before non-selective excitation. Gradient echoes were acquired with alternating readout gradient polarity at 6 equidistant times between 2.2ms and 14.7ms. For PD-weighted acquisition, two additional at 17.2ms and 19.7ms were acquired. A high readout bandwidth of 425Hz/pixel was used to reduce off-resonance artefacts. During acquisition subjects were encouraged to be as still as possible with eyes open or closed.

MRI data analysis

Functional imaging data analysis were carried out using SPM12. (http://www.fil.ion.ucl.ac.uk/spm/software/spm12/). After discarding the first three volumes to allow for magnetic saturation effects, the remaining images were realigned and unwarped to the first volume to correct for movement of subjects during scanning. Realigned images were then normalized to stereotactic space corresponding to the Montreal Neurological Institute “ICBM152” with parameters estimated from the structural scans and finally smoothed with a 3D Gaussian kernel of 6mm full-width at half maximum. After pre-processing the general linear model (GLM) was used for statistical analysis. The design matrix comprised events using a boxcar function

convolved with the canonical haemodynamic response function provided in SPM. Motion parameters estimated during the realignment step were included in the design matrix.

In the GLM analysis we modelled each of the three types of sound (trigger, unpleasant and neutral) as a separate event of duration 15s with silent periods as implicit baseline. The design matrix also included button presses and motion regressors as regressors of no-interest. A high pass filter with a cut off frequency of 1/128 Hz was applied to remove low frequency fluctuations in the BOLD signal. Once the GLM for each subject was estimated, whole-brain random effects analysis was implemented by entering contrasts of parameter estimates for each individual subject into second-level *F* and *t-tests.* Interaction between group and sound type was computed using a 2x3 ANOVA with group (Misophonic and Controls) and sound type (trigger, unpleasant and neutral) as factors, and subject effects modelled. Post hoc comparison of activity for simple effects between the two groups was done using two sample t-tests.

Functional connectivity analysis was performed using CONN toolbox [S5] (v 15.d). The time series was extracted from anatomically defined ROI (as given in SPM12, neuromorphometrics toolbox) of anterior insula. The effect of movement on the BOLD signal was reduced by regressing out motion parameters, along with their first order temporal derivative, by running whole brain voxel-wise regression. Additionally, five covariates generated using the aCmpCorr method [S6], which uses principal component analysis (PCA) on the measurements made in the white matter and CSF of each individual subjects segmented white matter and CSF masks, were used. The data were then high pass filtered with a cut-off frequency of 1/125 Hz. First-level functional connectivity for each group and condition was computed using bivariate correlation coefficient between the seed time series and time series from all other voxels in the brain. The 'neutral sounds' condition was taken as a baseline and comparison of connectivity between the two groups at second level was undertaken using two sample t-tests.

Structural data were analysed using voxel-based quantification (VBQ) toolbox [S1] in SPM12. Four quantitative maps (effective proton density, PD^*; longitudinal relaxation rate, R1; magnetization transfer saturation, MT and effective transverse relaxation rate, $R2^*$) were computed for each subject in the two groups. Briefly for MT, PD^* and R1 maps, the set of echoes for respective weightings were averaged to increase the signal to noise ratio and the resulting 3 volumes were subjected to the procedure as outlined in [S1,S7, S8] . To obtain $R2^*$ maps, log signal from the 8-PD weighted images was regressed against echo time. For further details of map creation, please see Callaghan et al (2014) [S9]. For performing voxel-based analysis, the MT maps (from each subject) were segmented into gray and white matter probability maps using the unified segmentation algorithm [S10]. Inter-subject co-registration of these tissue segments was performed using DARTEL, a nonlinear diffeomorphic algorithm as implemented in SPM12. This algorithm iteratively creates an average (across subjects) template and determines the deformations that best align the tissue maps to the average template. In our study average template was created by combining images from the two groups. The quantitative maps were co-registered, they were normalized to MNI space using subject specific deformations using a combined probability weighting and smoothing (Gaussian kernel of 3mm FWHM) procedure [S11]. This approach aims to preserve the quantitative values during the normalisation procedure by minimizing any partial volume effects introduced by the smoothing process. Statistically significant differences in tissues between the two groups were assessed using two sample t-tests implemented in SPM12. Age, sex and total intracranial cranial volume were included as regressors of no-interest. We also did a conventional voxel based morphometry (VBM) analysis on the cohort's MT maps, but this analysis did not reveal any significant differences. VBM analyses are dependent on many different parameters (algorithm, priors, and the contrast of the input) and interpretation of any findings, or lack thereof, is complex. Presumably the difference in MT values between the two groups, which could be identified via our quantitative comparison, was insufficient to greatly alter the segmentation result (since the expected MT value of adjacent white matter will be even higher still, e.g. reported as >1.7 in [S12] underpinning the VBM analysis.

Galvanic skin response and heart rate analysis

Galvanic skin response was recorded by placing two Ag/AgCl electrodes on the middle and ring fingers of non-dominant hand. The electrodes were connected to a custom built constant voltage (2.5 volt) coupler. The output of the voltage coupler was converted into an optical pulse frequency with an offset of 125Hz. The pulse signal was digitally recorded using Micro 1401/Spike2 (Cambridge Design) and then converted back to units of

conductance. Heart rate data was measured using a pulse oximeter connected on the index figure of the non-dominant hand and was digitally recorded using Micro 1401/Spike2. The amplifier encoding heart rate and GSR received TTL pulses from the scanner console identifying the current acquisition slice allowing synchronisation of physiological data to the fMRI data and experimental paradigm.

GSR data for 2 misophonic and 4 control subjects could not be used in the final analysis because of technical problems in recording data. Heart rate data for 2 control subjects could also not be used in the final analysis because of technical issues in data recording.

GSR data were first resampled to a sampling frequency of 10 Hz before filtering with a 2nd order Butterworth bandpass filter with cut-off frequencies [0.0015 2.5] Hz. The data were then epoched for each trial from 5s prior to sound onset of sound until 23s after onset. Data were visually inspected (blinded to trial type) and trials with artefacts were rejected. The trials were then sorted into different conditions (Trigger sounds/Unpleasant sounds/ Neutral sounds). After baseline (5s prior to onset of sound) correction, average evoked GSR across trials was computed. Statistical analysis was performed on the evoked GSR time series using a 2x3 ANOVA, as in the fMRI analysis. Correction for multiple comparison was done using cluster level thresholding implemented in SPM for M/EEG with a family wise error (FWE) threshold of $p < 0.05$ and cluster forming threshold of p=0.05. This method of correction finds periods of data where more contiguous data points than would be expected by chance pass the cluster forming threshold based upon estimation of the 1-dimensional smoothness inherent in the time series.

HR data were first converted into a continuous time series by inferring instantaneous heart rate based upon the interbeat interval and using spline interpolation with a supersampled 10Hz timecourse. The data were visually inspected for artefacts and periods with average HR < 45 bpm or > 120 bpm automatically rejected. Data were then epoched into trials, and baseline corrected as in the analysis of GSR data. After computing the evoked HR response, it was subjected to statistical analysis using the same procedure as for GSR data.

Mediation Analysis

We used whole brain single-level mediation analysis. Mediation analysis is a 3-variable path analysis [S13] which tests if the relationship between an input (X) and output variable (Y) is mediated by a third variable (M). It compares two models: a reduced model (equation 1) and a full model (equation 2)

$$Y = cX + e_y \qquad (1)$$

$$M = aX + e_M; \quad Y = bM + c'X + e_Y \qquad (2)$$

Mediation analysis tests if the difference:

$$c - c' = ab$$

is significantly different. If the difference is significant then it means that part (or whole) of the variance of Y which is explained by X alone (equation 1) can be explained by the mediator variable M (equation 2, second half) leading to a reduced value of c' compared to c.

In our study we asked which brain regions would explain higher heart rate and skin conductance in misophonic subjects compared to control subjects. For this analysis, our input variable X was a categorical variable (1 for the misophonic -1 for controls). The output variable Y was the average heart rate or skin conductance value for the subject. The mediator variable was the contrast (trigger sound-neutral sound) for each subject. The significance of mediation (ab) was tested using bias corrected bootstrap testing [S14] with 10000 samples at each voxel and the p-value at each voxel was calculated.

Questionnaire data analysis

To evaluate interoceptive sensibility [S15], the body consciousness questionnaire (BCQ) [S16] was administered after completion of the fMRI component of the study. Participants were requested to fill-in the questionnaire online. Nineteen misophonic and 14 control subjects filled in the questionnaire. The BCQ questionnaire has 15 questions in total with 5 questions in each of the three categories: Private Body Consciousness, Public Body Consciousness and Body Competence. The part of the BCQ related to private body evaluates perception of body as perceived from inside and comprises questions like: *I am sensitive to internal bodily tensions*; *I know immediately when my mouth or throat gets dry*. In public body consciousness, perception of body as visible to others is evaluated and consists of questions like: *when with others, I want my hands to be clean and look nice*; *I am aware of my best and worst facial features*. The body competence part of the BCQ evaluates awareness of the physical condition of body and includes questions like: *For my size, I am pretty strong; I am better coordinated than most people*. Each question has 5 options which are scored from 0 (Extremely uncharacteristic) to 4 (Extremely characteristic) thus making a maximum possible score of 60. Data were analysed by comparing the scores between misophonics and controls on the three categories separately using non-parametric equivalent to the two sample t-test (Mann-Whitney U test for unequal medians). Because of relatively small sample size of the control population who completed the questionnaire and to further check the reliability of our results, we also compared the BCQ scores of misophonic participants with scores of a larger population (n=136; 74 females, age range 25 to 63 years) of healthy controls on the same questionnaire. These data are a subset of data collected from healthy control participants by one of the co-authors (JSW) as a part of a separate study; the larger sample includes a large number of younger participants (n=208) who were excluded from this comparison to ensure average age matching between groups (t_{153}=0.5, p=0.61) although we checked that inclusion of the remaining younger control participants made no difference to the pattern of results). In order to further explore differences between misophonic participants and healthy controls, we also performed a type of case-control analysis, in which the scores for each patient were compared to the mean scores of healthy controls within this larger population matched for gender and within 3 years of age. The sign test was then used to assess whether participants with misophonia scored systematically differently from controls for the subscales of the questionnaire; the pattern of results was the same as for the more traditional analysis against a control population.

Supplemental References:

S1. Weiskopf, N., Suckling, J., Williams, G., Correia, M. M., Inkster, B. (2013). Quantitative multi-parameter mapping of R1 , PD* , MT , and R2* at 3T : a multi-center validation. Front. Neurosci. 7, *95*, 1-11.

S2. Weiskopf, N., Hutton, C., Josephs, O., Deichmann, R. (2006). Optimal EPI parameters for reduction of susceptibility-induced BOLD sensitivity losses: a whole-brain analysis at 3 T and 1.5 T. Neuroimage. *33*, 493–504.

S3. Lutti, A., Hutton, C., Finsterbusch, J., Helms, G., Weiskopf, N. (2010). Optimization and validation of methods for mapping of the radiofrequency transmit field at 3T. Magn. Reson. Med. *64*, 229–238.

S4. Lutti, A., Stadler, J., Josephs, O., Windischberger, C., Speck, O., Bernarding, J., Hutton, C., Weiskopf, N. (2012). Robust and fast whole brain mapping of the RF transmit field B1 at 7T. PLoS One. *7*, e32379.

S5. Whitfield-Gabrieli, S., Nieto-Castanon, A. (2012). Conn: a functional connectivity toolbox for correlated and anticorrelated brain networks. Brain. Connect. *2*, 125–141.

S6. Behzadi, Y., Restom, K., Liau, J., Liu, T. T. (2007). A component based noise correction method (CompCor) for BOLD and perfusion based fMRI. Neuroimage. *37*, 90–101.

S7. Helms, G., Dathe, H., Dechent, P. (2008). Quantitative FLASH MRI at 3T using a rational approximation of the Ernst equation. Magn. Reson. Med. *59*, 667–672.

S8. Helms, G., Dathe, H., Kallenberg, K., Dechent, P. (2008). High-resolution maps of magnetization transfer with inherent correction for RF inhomogeneity and T1 relaxation obtained from 3D FLASH MRI. Magn. Reson. Med. *60*, 1396–1407.

S9. Callaghan, M. F., Freund, P., Draganski, B., Anderson, E., Cappelletti, M., Chowdhury, R., Diedrichsen, J., Fitzgerald, T. H., Smittnear, P., Helms, G. et. al. (2014). Widespread age-related differences in the human brain microstructure revealed by quantitative magnetic resonance imaging. Neurobiol. Aging. *35*,

1862–1872.

S10. Ashburner, J., Friston, K. J. (2005). Unified segmentation. Neuroimage. *26,* 839–851.

S11. Draganski, B., Ashburner, J., Hutton, C., Kherif, F., Frackowiak, R. S. J., Helms, G., Weiskopf, N. (2011). Regional specificity of MRI contrast parameter changes in normal ageing revealed by voxel-based quantification (VBQ). Neuroimage. *55,* 1423–1434.

S12. Weiskopf, N., Suckling, J., Williams, G., Correia, M. M., Inkster, B., Tait, R., Ooi, C., Bullmore, E. T., Lutti, A. (2013). Quantitative multi-parameter mapping of R1 , PD*, MT, and R2* at 3T : a multi-center validation. Front. Neurosci. *7,* 1–11.

S13. Baron, R. M., Kenny, D. A. (1986). The moderator-mediator variable distinction in social psychological research: Conceptual, strategic and social considerations. J. Pers. Soc. Psychol. *51*, 1173–82.

S14. Efron, B., Tibshirani, R. (1993). An Introduction to the Bootstrap. (New York: Chapman & Hall/CRC Press).

S15. Garfinkel, S. N., Tiley, C., O'Keeffe, S., Harrison, N. A., Seth, A. K., Critchley, H. D. (2015) Discrepancies between dimensions of interoception in Autism: Implications for emotion and anxiety. Biol. Psychol. *114,* 117-126.

S16. Miller, L. C., Murphy, R., Buss, A. H. (1981). Consciousness of body: Private and public. J. Pers. Soc. Psychol. *41*, 397–406.

Supplemental Misophonia Questionaire S1

Name : ____________________________________

Age : ____________________________________

Sex: ______________

Q1: Please list the sounds that you dislike most (the best trigger sound first)

Q2: Does it make a difference who is making these sounds? (e.g. whether a close friend or family member makes them, or a stranger). If 'yes', please explain how it affects your reaction.

Q3: Could you plz rate on a scale, from 1 to 10 (1: no effect; 10 = maximum effect), your reaction when the trigger sound is produced by

i) A stranger _________________

ii) A close family member _________________

Q4: If we have to produce a strong trigger in our lab (in a MRI scanner for example) so that your brain activity under that condition could be monitored, do you think it could be done if

i. The recorded sounds alone, of the person who triggers strong reaction, are played back to you
ii. Just a picture of the person is shown.
iii. A silent video of the person doing the action that acts as a trigger (e.g. eating, breathing) is played.
iv. Both sounds and picture of the person are used.
v. Video with sounds is used

Please rate each of the above option on a scale from 1 to 10 (1 = no effect; 10= maximum effect)

Q5: Does the situation you are in make a difference to your reaction to these sounds? (e.g. whether you are at work, enjoying leisure activities or trying to relax). If 'yes', please explain how the situation affects your reaction.

Q6: Does the noise level around you affect your reaction to these sounds? (i.e. do you have more or less of a reaction in noisy surroundings).

Q7: If you can, please explain what it is about these sounds that you dislike.

Q8: Please describe the feeling you get when you hear these particular sounds.

Q9: Please describe what you do when you hear these sounds.

Q10: Please describe any steps you have taken to avoid hearing these sounds.

Q11: Please describe the effect that having misophonia has had on your life (including effects on employment, study, hobbies, social activities and relationship with friends and family).

Q12: When did you first notice these symptoms of strongly disliking certain sounds? Was there any particular event or trigger associated with the symptoms starting?

Q13: Have your symptoms changed over time since they began? If so, please explain in what way they have changed (e.g. getting worse, getting better, the reaction itself changing).

Q14: Please list any sounds that you particularly like.

Q15: Do you often experience tinnitus (ringing in the ears)? If so, how much of the time do you hear it, how loud is it (on a scale of 0-10) and how much does it bother you (on a scale of 1-10)?

Q16: As well as particularly disliking particular sounds, do you have a strong dislike of loud sounds in general (ones that other people around you do not seem to mind)?

Q17: Does anybody in your family have similar symptoms to yours? If so, please give details.

Q18: Do you have hearing loss? Have you had a hearing test? If so, what was the result?

Q19: Would you be happy if we contacted you in future to discuss taking part in research on misophonia which requires brain imaging (fMRI or MEG)? If so, please state your preferred contact details (e.g. e-mail address, phone number, postal address).

Q20 . Please provide any additional information.
