# Supplementary material for: The Brain Basis for Misophonia
Source: Curr Biol. 2017 Feb 20;27(4):527–33. doi: 10.1016/j.cub.2016.12.048 (PMC5321671; doi:10.1016/j.cub.2016.12.048)
Supplement: Document S2. Article plus Supplemental Information [file mmc2.pdf]

Report

Current Biology

The Brain Basis for Misophonia

Highlights

- Trigger sounds elicit exaggerated response in anterior insula in misophonia
- In misophonia, there is abnormal functional connectivity of anterior insula
- Heightened autonomic responses are mediated by anterior insula in misophonia
- Misophonia is associated with altered interoception

Authors

Sukhbinder Kumar, Olana Tansley-Hancock, William Sedley, ..., Phillip E. Gander, Doris-Eva Bamiou, Timothy D. Griffiths

Correspondence

sukhbinder.kumar@ncl.ac.uk

In Brief

Kumar et al. show that misophonia is associated with abnormal activation, functional connectivity, and structural changes in the brain and heightened autonomic responses of the body.

Kumar et al., 2017, Current Biology *27*, 527–533
February 20, 2017 © 2016 The Author(s). Published by Elsevier Ltd.
http://dx.doi.org/10.1016/j.cub.2016.12.048

Current Biology
Report

CellPress

The Brain Basis for Misophonia

Sukhbinder Kumar,[1,2,7,*] Olana Tansley-Hancock,[1] William Sedley,[1] Joel S. Winston,[2,3] Martina F. Callaghan,[2] Micah Allen,[2] Thomas E. Cope,[1,4] Phillip E. Gander,[5] Doris-Eva Bamiou,[6] and Timothy D. Griffiths[1,2]
[1]Institute of Neuroscience, Medical School, Newcastle University, Newcastle upon Tyne NE2 4HH, UK
[2]Wellcome Trust Centre for Neuroimaging, 12 Queen Square, London WC1N 3BG, UK
[3]Institute of Cognitive Neuroscience, 17 Queen Square, London WC1N 3AR, UK
[4]Department of Clinical Neurosciences, University of Cambridge, Cambridge CB2 0SZ, UK
[5]Human Brain Research Laboratory, Department of Neurosurgery, The University of Iowa, Iowa City, IA 52242, USA
[6]UCL Ear Institute, 332 Grays Inn Road, London WC1X 8EE, UK
[7]Lead Contact
*Correspondence: sukhbinder.kumar@ncl.ac.uk
http://dx.doi.org/10.1016/j.cub.2016.12.048

SUMMARY

Misophonia is an affective sound-processing disorder characterized by the experience of strong negative emotions (anger and anxiety) in response to everyday sounds, such as those generated by other people eating, drinking, chewing, and breathing [1–8]. The commonplace nature of these sounds (often referred to as "trigger sounds") makes misophonia a devastating disorder for sufferers and their families, and yet nothing is known about the underlying mechanism. Using functional and structural MRI coupled with physiological measurements, we demonstrate that misophonic subjects show specific trigger-sound-related responses in brain and body. Specifically, fMRI showed that in misophonic subjects, trigger sounds elicit greatly exaggerated blood-oxygen-level-dependent (BOLD) responses in the anterior insular cortex (AIC), a core hub of the "salience network" that is critical for perception of interoceptive signals and emotion processing. Trigger sounds in misophonics were associated with abnormal functional connectivity between AIC and a network of regions responsible for the processing and regulation of emotions, including ventromedial prefrontal cortex (vmPFC), posteromedial cortex (PMC), hippocampus, and amygdala. Trigger sounds elicited heightened heart rate (HR) and galvanic skin response (GSR) in misophonic subjects, which were mediated by AIC activity. Questionnaire analysis showed that misophonic subjects perceived their bodies differently: they scored higher on interoceptive sensibility than controls, consistent with abnormal functioning of AIC. Finally, brain structural measurements implied greater myelination within vmPFC in misophonic individuals. Overall, our results show that misophonia is a disorder in which abnormal salience is attributed to particular sounds based on the abnormal activation and functional connectivity of AIC.

RESULTS AND DISCUSSION

fMRI data were acquired in 20 misophonic and 22 age- and sex-matched controls while they listened to a set of three sounds: trigger sounds (which evoke a misophonic reaction in misophonic individuals; e.g., eating, breathing sounds), unpleasant sounds (which are perceived to be annoying by both groups but do not evoke misophonic distress; e.g., baby cry, a person screaming), and neutral sounds (e.g., rain). After listening to each sound, subjects rated (1) how annoying the sound was (both groups) and (2) how effectively the sound triggered a typical misophonic reaction (misophonic group only) or how anti-social (in the sense the subject would not like to be in the environment in which the sound is produced) the sounds were (control group only). Behavioral responses, galvanic skin response (GSR) and heart rate (HR), were acquired during the acquisition of fMRI data (see Figure 1A for a schematic of the paradigm). Whole-brain structural MRI data were acquired as multi-parameter maps (MPMs) [9] to measure myelination content, water, and iron levels.

Behavioral data (Figure 1B) showed that trigger sounds evoked misophonic distress in misophonic subjects, whereas the unpleasant sounds, although annoying, did not produce a misophonic reaction. There was no difference between the misophonic distress ratings of trigger sounds by the misophonic group and annoyance ratings of unpleasant sounds by the control group. It is likely, however, that the two groups used different subjective scales while rating the sounds. Random-effects analysis of fMRI data using the general linear model (GLM) [10] with group (two levels) and sound types (three categories) as factors demonstrated an interaction in the anterior insular cortex (AIC) bilaterally (Figure 2A; further regions are listed in Table S1). Further analysis showed that the interaction in AIC was driven by greater activation in misophonic subjects compared to control subjects in response to trigger sounds (see Figure 2B and Figure S1 for confirmatory plots; see also Figure S2). Significant activation differences between misophonic and control subjects did not occur to unpleasant or neutral sounds. Activity in both the left and right AIC varied linearly with the subjective rating of misophonic distress in the misophonic group, as shown in confirmatory plots in Figure 2C. A large body of evidence [11] implicates AIC in subjective feelings associated with emotions, including anger. Functionally, AIC is known to be a key node of

This is an open access article under the CC BY license (http://creativecommons.org/licenses/by/4.0/).

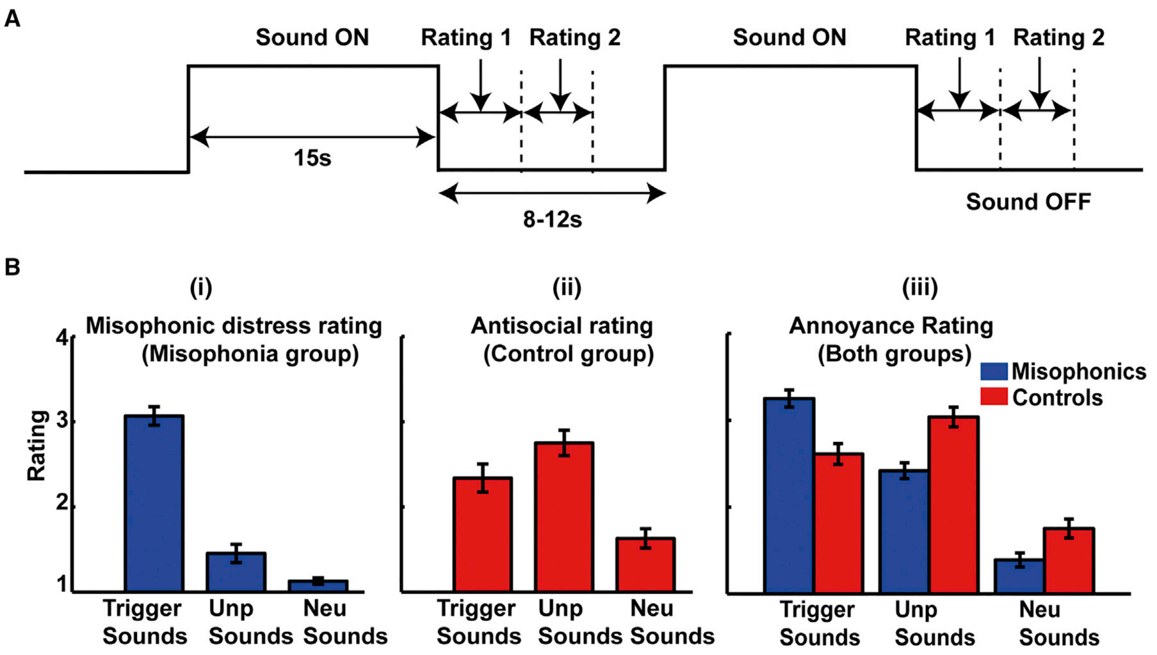

Figure 1. Experimental Paradigm and Subjective Ratings

(A) fMRI paradigm: a standard block design was used in which sounds were presented for 15 s. After every sound, subjects gave two ratings on a scale from 1 to 4 with a button press for (1) how annoying the sound was and (2) how effective the sound was in triggering misophonic reaction (misophonia group) or how antisocial the sound was (control group). fMRI data were acquired continuously with a repetition time (TR) of 3.12 s. GSR and HR were also monitored throughout the experiment.

(B) Subjective ratings: (i) misophonic distress rating of three types of sounds by misophonic group; (ii) antisocialness rating of sounds (control subjects); and (iii) annoyance rating of sounds by both groups. Misophonic subjects rated the trigger sounds as evoking greater misophonic reaction compared to unpleasant ($p < 0.001$) and neutral sounds ($p < 0.001$). Unpleasant sounds were still perceived to be annoying ($p < 0.001$ compared to neutral sounds) by the misophonic subjects, demonstrating a dissociation between general annoyance and misophonic reaction. See also Figure S4 for subjective scores on body perception. Data are represented as mean (±SEM).

the salience network [12], an intrinsic large-scale brain network for detecting and orienting attention toward stimuli that are behaviorally relevant and meaningful for an individual. Specific hyperactivity in AIC to trigger sounds supports the hypothesis that misophonic subjects assign aberrantly higher salience to these sounds.

Having identified AIC as a key region that differentiates trigger sounds in misophonic participants, we sought to explore its stimulus-dependent connectivity profile to establish whether there are alterations at the network level that are specific to misophonia. Using left AIC as a seed region, we analyzed its stimulus-dependent connectivity in the two groups. Greater functional connectivity of AIC for misophonic subjects was observed in a network of brain regions comprising the ventromedial prefrontal cortex (vmPFC), posteromedial cortex (PMC; posterior cingulate and retrosplenial cortex), hippocampus, and amygdala (Figure 3A). This increased functional connectivity was specific to trigger sounds: no significant differences in connectivity were observed for unpleasant sounds. Importantly, the functional connectivity pattern between the two groups for the same sounds was not only different quantitatively but also qualitatively: whereas the connectivity to vmPFC is positive (with respect to connectivity for neutral sounds) in misophonic subjects, the connectivity for controls for the same set of sounds is negative. Analysis of functional connectivity of right AIC also showed trigger-sound-specific increased connectivity to vmPFC and PMC (Figure S3A; functional connectivity to amygdala and hippocampus was also observed but at a slightly relaxed threshold). The vmPFC and PMC together form core parts of the default mode network (DMN) [13] (see Figure S3B for overlap between the DMN and the functional connectivity network of AIC), which is activated when subjects are engaged in internally directed thoughts and retrieval of memories [14] and is deactivated when attention is directed to external stimuli. Greater coupling of AIC with the DMN suggests that misophonic subjects, on hearing trigger sounds, are unable to “disengage” AIC from the DMN, which entails memories and contextual associations of trigger sounds to bear on the activation of AIC. This is also consistent with a recent study [15] using multivariate pattern classification, which showed that patterns of activity in vmPFC and PMC were most informative in distinguishing different types of emotions. Distinct functional connectivity of AIC to vmPFC and PMC in misophonics and controls for the same sounds suggests that these regions play a crucial role in instantiating different emotional responses for the trigger sounds in the two groups. This atypical functional connectivity could, therefore, underlie the abnormal activation of AIC and the aberrant salience assigned to trigger sounds by the misophonic group.

Because misophonia symptoms start early in life (mean age of onset is ~12 years and can be as early as 5 years [1]), we also

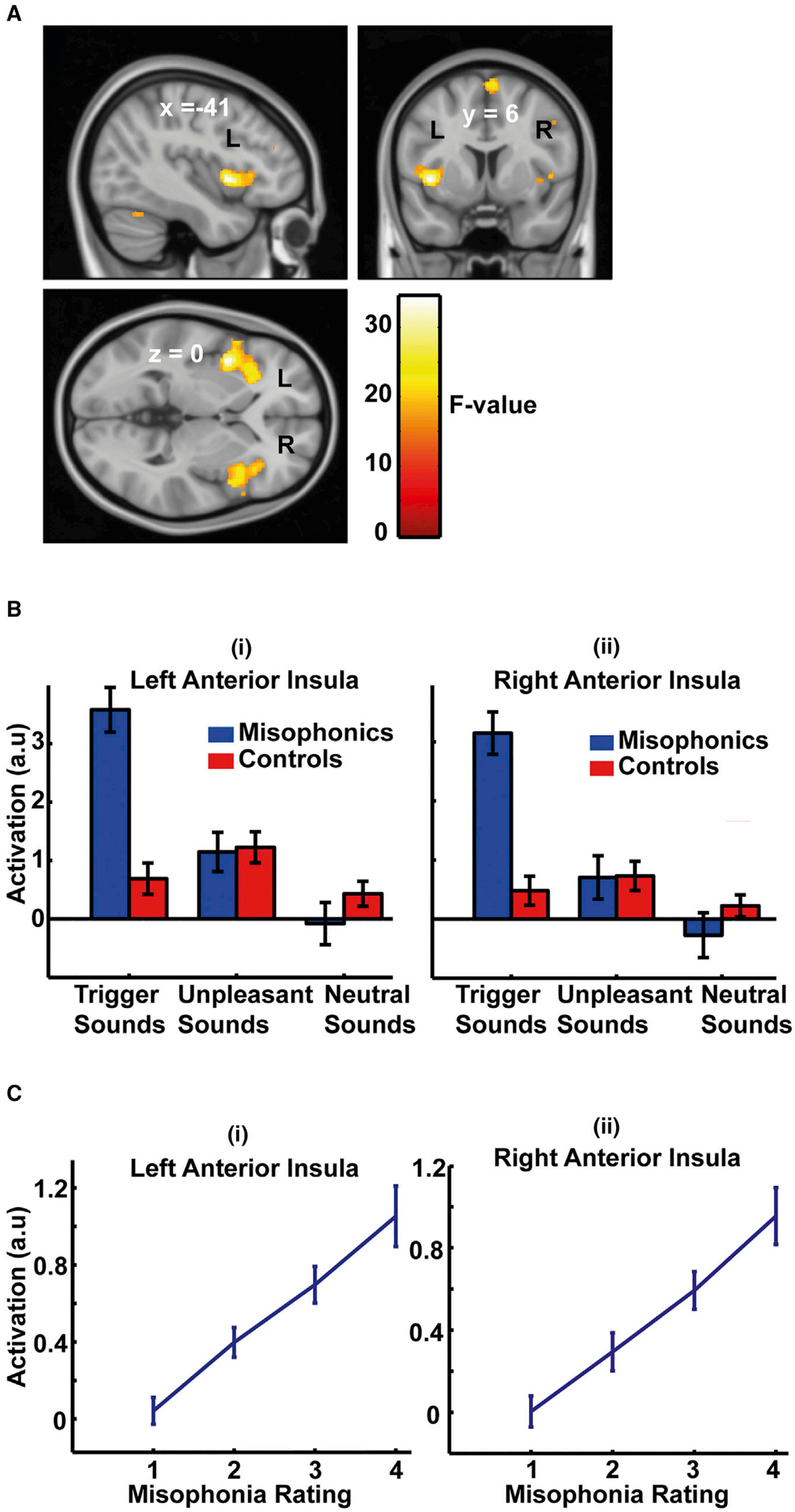

Figure 2. Group-Level, Random-Effects GLM Analysis of fMRI Data

The GLM was modeled as a factorial design with group (two levels) and sound types (three levels) as factors.

(A) Statistical parameter maps (SPMs) overlaid on a standard MNI-152 template brain for the critical interaction between the two factors (group and sound type) thresholded at p = 0.05 family-wise error (FWE) corrected for whole-brain volume. The effect is maximal in AIC (bilateral) with maxima at MNI coordinates (−41, 6, 0).

(B) Confirmatory plots of activity averaged over cluster in AIC (see also Figures S1 and S2 and Table S1) show that the interaction effect was driven by higher activity for trigger sounds in misophonic subjects compared to controls.

(C) Confirmatory plots of activity in AIC with misophonic ratings in misophonic subjects.

Data in (B) and (C) show mean (± SEM).

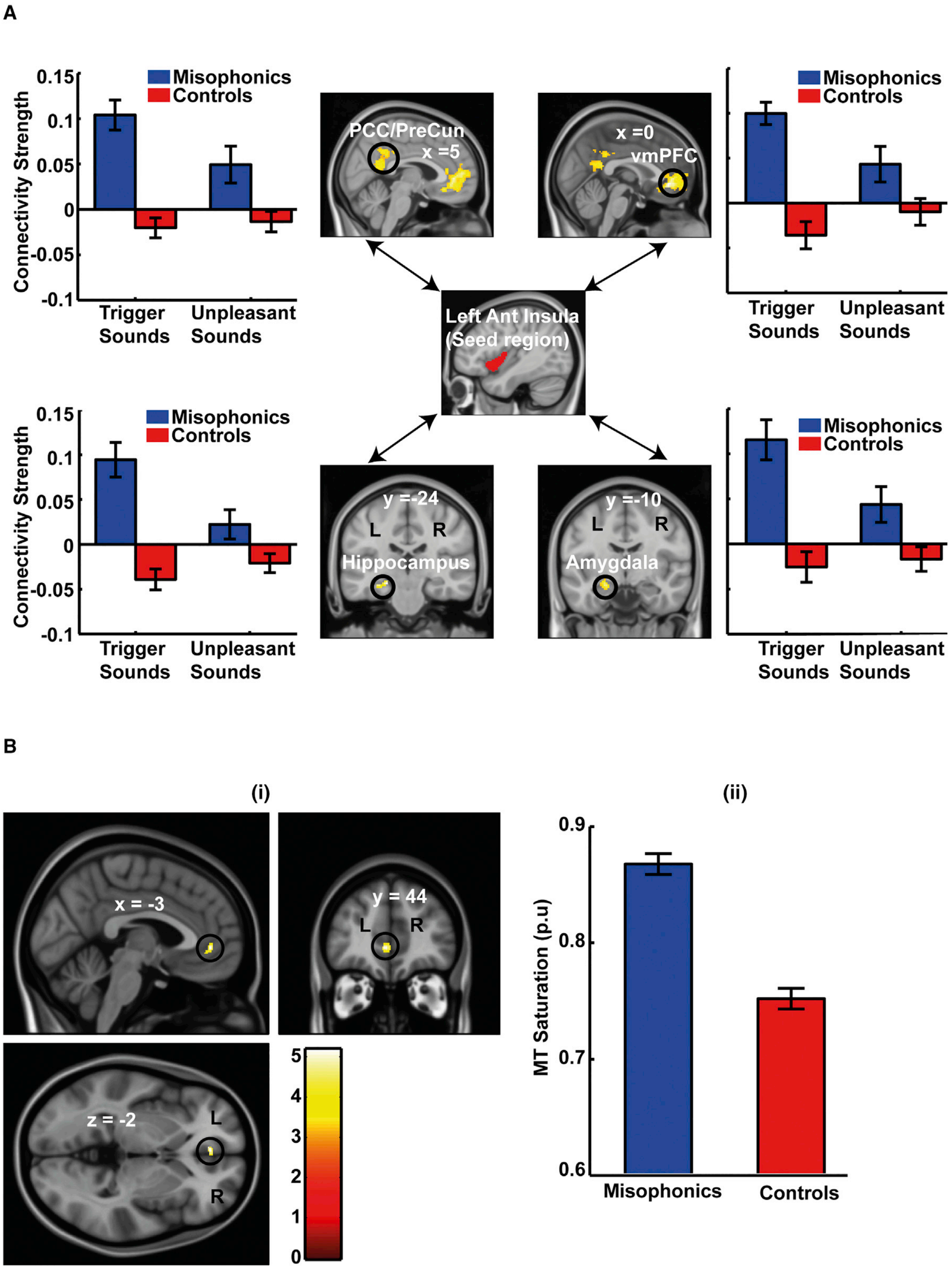

Figure 3. Functional Connectivity and Structural Data Analysis

(A) Left AIC was taken as a seed region and its functional connectivity to all voxels of the brain was analyzed. The figure illustrates those brain areas that show greater connectivity for trigger sounds (compared to neutral sounds) in misophonic subjects (compared to controls). The four areas that survive the threshold are (1) PMC (posterior cingulate cortex [PCC]/precuneus), (2) vmPFC, (3) hippocampus, and (4) amygdala. The bar chart for each region shows confirmatory plots of connectivity for trigger and unpleasant sounds with respect to neutral sounds. Displayed connectivity strengths are cluster thresholded at $p < 0.05$ with cluster-forming threshold at $p < 0.001$ (see Figure S3 for functional connectivity of right AIC and overlap of the connectivity network with the default mode network).

(legend continued on next page)

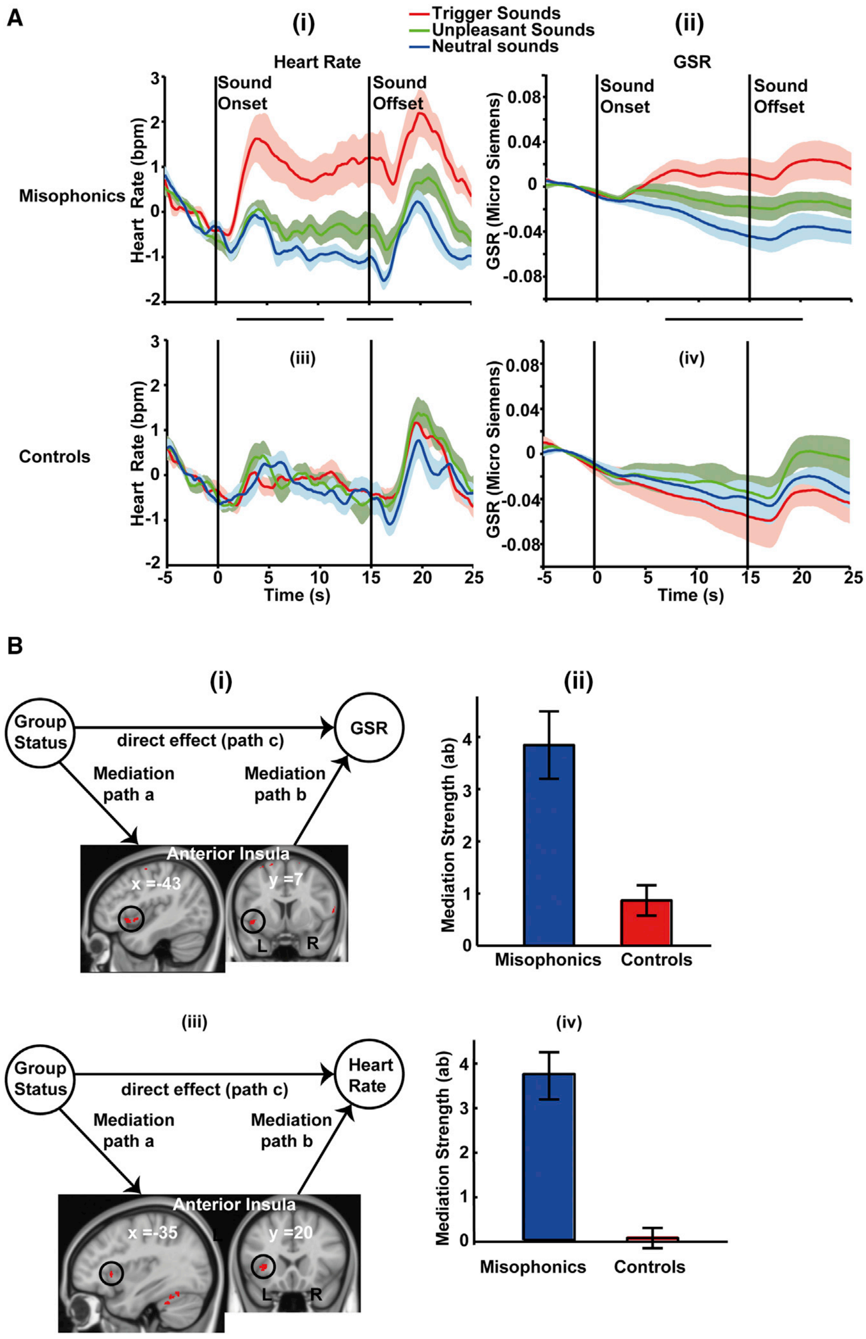

Figure 4. Psychophysiological Responses and Their Mediation by Brain Areas

(A) HR and GSR for misophonic and control subjects. In misophonic subjects, the trigger sounds produce sustained increases in HR and GSR. Statistical analysis of GSR and HR was performed time by time using a 2 × 3 ANOVA as in the fMRI analysis. For the HR time series, interaction between the factors was significant from 2.4 to 10.4 s and then from 12.4 to 17 s after sound onset. For the GSR time series, significant interaction was observed from 7 to 21.4 s after sound onset (time points at which GSR and HR are significantly different are indicated by black horizontal bars between the panels). Both HR and GSR time series were cluster thresholded at $p < 0.05$ with cluster-forming threshold at $p < 0.05$. Post hoc comparison showed that the interaction effect in both HR and GSR was driven by higher responses to trigger sounds in misophonic subjects. There was no difference between the two groups in their responses to unpleasant and neutral sounds. bpm, beats per min.

(B) Mediation analysis to determine which brain areas mediate the increased HR and GSR in misophonic subjects, relative to controls, to trigger sounds. Whole-brain, single-level mediation analysis was used, in which the input X is a categorical vector (+1 for misophonics and −1 for controls) and the response vector Y contains an average increase in HR/GSR (compared to neutral sounds) over a trial of trigger sounds for each subject. The mediation variable M is the beta value (as determined using SPM) for trigger sounds compared to neutral sounds. (i) Left AIC mediates GSR changes. (ii) Confirmatory plots of mediation strength for GSR for the two groups averaged over the cluster in AIC. (iii) AIC mediates heightened HR in misophonics. (iv) Confirmatory plots of mediation strength for HR for the two groups averaged over the cluster in AIC. The displayed results (i) and (iii) are thresholded at $p < 0.005$ with a cluster extent threshold of 50 voxels.

Data are represented as mean (± SEM; shaded areas in A and error bars in B).

predicted that there would be brain structural differences in misophonic subjects compared to controls. We created whole-brain structural maps of magnetization transfer (MT) saturation that reflects myelination in brain gray matter. For significance testing, we limited our search to brain areas that showed higher functional connectivity to AIC in misophonics compared to controls along with the seed region. Analysis of structural maps showed that misophonic subjects have altered MT saturation, which is consistent with significantly higher myelination in the gray matter of vmPFC (Figure 3B). This change suggests a possible structural basis for the altered functional connectivity to vmPFC observed in misophonic subjects.

After identification of functional and structural changes in the brain, we next determined physiological responses of the body

(B) Brain structural changes in misophonia. Misophonic subjects show higher MT saturation, which reflects higher myelination, compared to controls in vmPFC. When corrected for multiple comparisons ($p < 0.05$ FWE corrected for brain areas that show higher functional connectivity in misophonics to trigger sounds; i.e., the functional network shown in (A) along with the seed region AIC), 15 voxels of vmPFC with maxima at (−3, 44, −2) survive the correction. For display purposes in the figure, a threshold of $p < 0.001$ uncorrected is used. p.u., percent units.

Data in bar charts show mean (± SEM).

and their driving sources in the brain. We measured GSR and HR while subjects listened to three sets of sounds in the MRI scanner. Trigger sounds evoked greater GSR and HR responses in misophonic subjects than control subjects (Figure 4A). Physiological responses were sustained throughout the duration of sound presentation and were specific to trigger sounds, with no difference in GSR or HR response between the two groups for unpleasant and neutral sounds. The heightened trigger-specific autonomic responses we observed are consistent with the strong tendency of misophonic subjects to escape from the environment of trigger sounds [1, 2] or experience strong anxiety and anger if unable to escape (fight/flight response).

What is the brain source(s) of these heightened autonomic responses in misophonia? To answer this, we used mediation analysis [16], which aims to test whether a relation from variable X (group membership; i.e., misophonic or control) to Y (GSR or HR) could be explained (mediated) by a third variable, M (brain activation). A significant mediation implies that there is an indirect path to Y (X to M to Y) and would show brain activity (M) that can mediate the observed GSR/HR (Y) over and above what is explained by group membership (X). We ran the whole-brain mediation analysis separately for GSR and HR. We found that activity in AIC mediated both the heightened GSR and HR (Figure 4B) in misophonic subjects.

Over the last decade, there has been a growing recognition that interoception (perception of internal bodily states) can influence the salience and experience of emotions associated with a stimulus [17–20]. Interestingly, AIC is the key brain structure that integrates ascending visceral inputs from the body with external sensory inputs. In accordance with this, atypical interoception and activation in AIC have been shown to underlie a number of social-emotional disorders [21, 22]. Recently, there has been a growing interest in extending prediction-based hierarchical Bayesian inference as a model of interoception [19, 23]. In this model, interoception involves inferring causes of interoceptive signals by combining bottom-up interoceptive signals with prior beliefs (predictions) of their causes. In this multi-level and hierarchically organized inference scheme, AIC is at the top of the hierarchy and is suggested to infer the overall state of the body [24]. Evaluation of subjective beliefs about body perception using the Body Consciousness Questionnaire [25] showed that misophonics report greater awareness of internal sensations (Figure S4) compatible with altered interoceptive sensibility [22] in misophonics. Given the role of AIC in representing bodily states, the questionnaire data are also consistent with abnormal AIC functioning in misophonia.

Conclusions

Overall, our data show that for misophonics, trigger sounds cause hyperactivity of AIC and an abnormal functional connectivity of this region with medial frontal, medial parietal, and temporal regions; that there is abnormal myelination in medial frontal cortex that shows abnormal functional connectivity to AIC; and that the aberrant neural response mediates the emotional coloring and physiological arousal that accompany misophonic experiences. Together, our data suggest that abnormal salience attributed to otherwise innocuous sounds, coupled with atypical perception of internal body states, underlies misophonia. With the available data, it is not possible to decide whether misophonia is a cause or consequence of atypical interoception, and further work is needed to delineate the relation between the two.

Misophonia does not feature in any neurological or psychiatric classification of disorders; sufferers do not report it for fear of the stigma that this might cause, and clinicians are commonly unaware of the disorder. This study defines a clear phenotype based on changes in behavior, autonomic responses, and brain activity and structure that will guide ongoing efforts to classify and treat this pernicious disorder.

SUPPLEMENTAL INFORMATION

Supplemental Information includes Supplemental Experimental Procedures, four figures, one table, and one questionnaire and can be found with this article online at http://dx.doi.org/10.1016/j.cub.2016.12.048.

AUTHOR CONTRIBUTIONS

S.K., O.T.-H., W.S., J.S.W., and T.D.G. designed the experiments; S.K. and O.T.-H. collected the data; S.K., with help from W.S., J.S.W., M.F.C., and T.D.G., analyzed the data; S.K., O.T.-H., W.S., J.S.W., M.F.C., M.A., T.E.C., P.E.G., D.-E.B., and T.D.G. wrote the paper; and T.D.G. supervised all aspects of the work.

ACKNOWLEDGMENTS

T.D.G. would like to thank the Wellcome Trust for the financial support of this project (grants WT091681MA and WT106964). J.S.W. holds a Wellcome Trust Postdoctoral Training Fellowship for MB/PhD Graduates (grant 095939). The Wellcome Trust Centre for Neuroimaging is supported by core funding from the Wellcome Trust (grant 091593). This study was approved by the UCL ethics committee.

Received: September 20, 2016
Revised: November 21, 2016
Accepted: December 22, 2016
Published: February 2, 2017

REFERENCES

1. Kumar, S., Hancock, O., Cope, T., Sedley, W., Winston, J., and Griffiths, T.D. (2014). Misophonia: a disorder of emotion processing of sounds. J. Neurol. Neurosurg. Psychiatry *85*, e3.
2. Edelstein, M., Brang, D., Rouw, R., and Ramachandran, V.S. (2013). Misophonia: physiological investigations and case descriptions. Front. Hum. Neurosci. *7*, 296.
3. Schröder, A., Vulink, N., and Denys, D. (2013). Misophonia: diagnostic criteria for a new psychiatric disorder. PLoS ONE *8*, e54706.
4. Schröder, A., van Diepen, R., Mazaheri, A., Petropoulos-Petalas, D., Soto de Amesti, V., Vulink, N., and Denys, D. (2014). Diminished N1 auditory evoked potentials to oddball stimuli in misophonia patients. Front. Behav. Neurosci. *8*, 123.
5. Jastreboff, M.M., and Jastreboff, P.J. (2001). Components of decreased sound tolerance: hyperacusis, misophonia, phonophobia. ITHS News Lett. *2*, 5–7.
6. Krauthamer, J.T. (2013). Sound-Rage: A Primer of the Neurobiology and Psychology of a Little Known Anger Disorder (Chalcedony Press).
7. Hadjipavlou, G., Baer, S., Lau, A., and Howard, A. (2008). Selective sound intolerance and emotional distress: what every clinician should hear. Psychosom. Med. *70*, 739–740.
8. Ferreira, G.M., Harrison, B.J., and Fontenelle, L.F. (2013). Hatred of sounds: misophonic disorder or just an underreported psychiatric symptom? Ann. Clin. Psychiatry *25*, 271–274.
9. Weiskopf, N., Suckling, J., Williams, G., Correia, M.M., Inkster, B., Tait, R., Ooi, C., Bullmore, E.T., and Lutti, A. (2013). Quantitative multi-parameter

mapping of R1, PD(*), MT, and R2(*) at 3T: a multi-center validation. Front. Neurosci. *7*, 95.

10. Friston, K.J., Holmes, A.P., Worsley, K.J., Poline, J.P., Frith, C.D., and Frackowiak, R.S.J. (1994). Statistical parametric maps in functional imaging: a general linear approach. Hum. Brain Mapp. *2*, 189–210.
11. Craig, A.D. (2009). How do you feel—now? The anterior insula and human awareness. Nat. Rev. Neurosci. *10*, 59–70.
12. Seeley, W.W., Menon, V., Schatzberg, A.F., Keller, J., Glover, G.H., Kenna, H., Reiss, A.L., and Greicius, M.D. (2007). Dissociable intrinsic connectivity networks for salience processing and executive control. J. Neurosci. *27*, 2349–2356.
13. Raichle, M.E., MacLeod, A.M., Snyder, A.Z., Powers, W.J., Gusnard, D.A., and Shulman, G.L. (2001). A default mode of brain function. Proc. Natl. Acad. Sci. USA *98*, 676–682.
14. Huijbers, W., Pennartz, C.M., Cabeza, R., and Daselaar, S.M. (2011). The hippocampus is coupled with the default network during memory retrieval but not during memory encoding. PLoS ONE *6*, e17463.
15. Saarimäki, H., Gotsopoulos, A., Jääskeläinen, I.P., Lampinen, J., Vuilleumier, P., Hari, R., Sams, M., and Nummenmaa, L. (2016). Discrete neural signatures of basic emotions. Cereb. Cortex *26*, 2563–2573.
16. Wager, T.D., Waugh, C.E., Lindquist, M., Noll, D.C., Fredrickson, B.L., and Taylor, S.F. (2009). Brain mediators of cardiovascular responses to social threat: part I: reciprocal dorsal and ventral sub-regions of the medial prefrontal cortex and heart-rate reactivity. Neuroimage *47*, 821–835.
17. Craig, A.D. (2007). Interoception and emotion: a neuroanatomical perspective. In Handbook of Emotions, M. Lewis, J.M. Haviland-Jones, and L.F. Barett, eds. (Guilford Press), pp. 272–290.
18. Wiens, S. (2005). Interoception in emotional experience. Curr. Opin. Neurol. *18*, 442–447.
19. Seth, A.K. (2013). Interoceptive inference, emotion, and the embodied self. Trends Cogn. Sci. *17*, 565–573.
20. Garfinkel, S.N., and Critchley, H.D. (2013). Interoception, emotion and brain: new insights link internal physiology to social behaviour. Commentary on: "Anterior insular cortex mediates bodily sensibility and social anxiety" by Terasawa et al. (2012). Soc. Cogn. Affect. Neurosci. *8*, 231–234.
21. Paulus, M.P., and Stein, M.B. (2010). Interoception in anxiety and depression. Brain Struct. Funct. *214*, 451–463.
22. Garfinkel, S.N., Tiley, C., O'Keeffe, S., Harrison, N.A., Seth, A.K., and Critchley, H.D. (2016). Discrepancies between dimensions of interoception in autism: implications for emotion and anxiety. Biol. Psychol. *114*, 117–126.
23. Seth, A.K., and Friston, K.J. (2016). Active interoceptive inference and the emotional brain. Philos. Trans. R. Soc. Lond. B Biol. Sci. *371*, 20160007.
24. Smith, R., and Lane, R.D. (2015). The neural basis of one's own conscious and unconscious emotional states. Neurosci. Biobehav. Rev. *57*, 1–29.
25. Miller, L.C., Murphy, R., and Buss, A.H. (1981). Consciousness of body: private and public. J. Pers. Soc. Psychol. *41*, 397–406.

Current Biology, Volume *27*

Supplemental Information

The Brain Basis for Misophonia

Sukhbinder Kumar, Olana Tansley-Hancock, William Sedley, Joel S. Winston, Martina F. Callaghan, Micah Allen, Thomas E. Cope, Phillip E. Gander, Doris-Eva Bamiou, and Timothy D. Griffiths

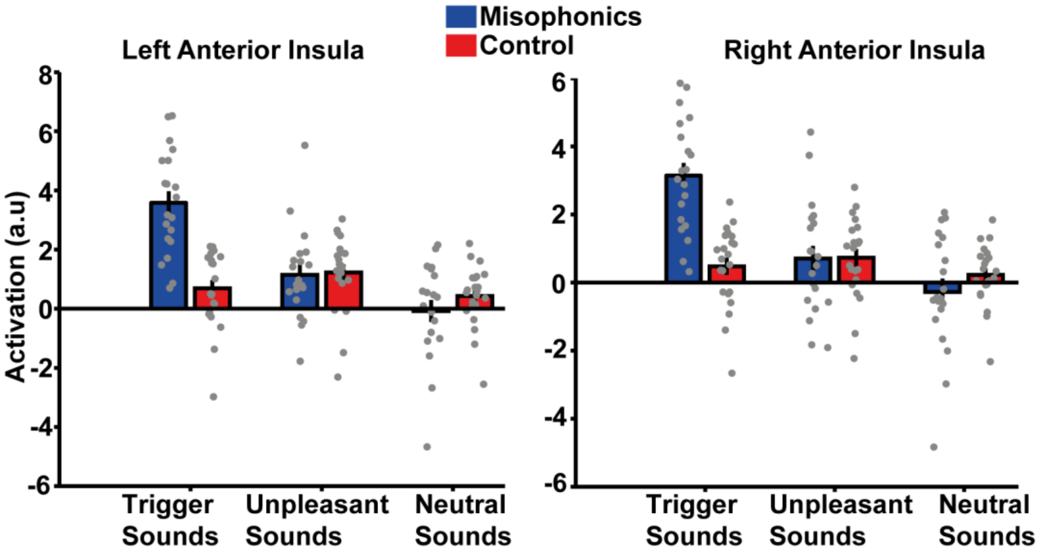

Figure S1: **Mean and distribution of anterior insula response; Related to Figure 2**. Plots of individual subject data along with the mean and standard error for activation (as determined by general linear model analysis) in the left and right anterior insula for both groups. Data in the bars are represented as mean (± SEM).

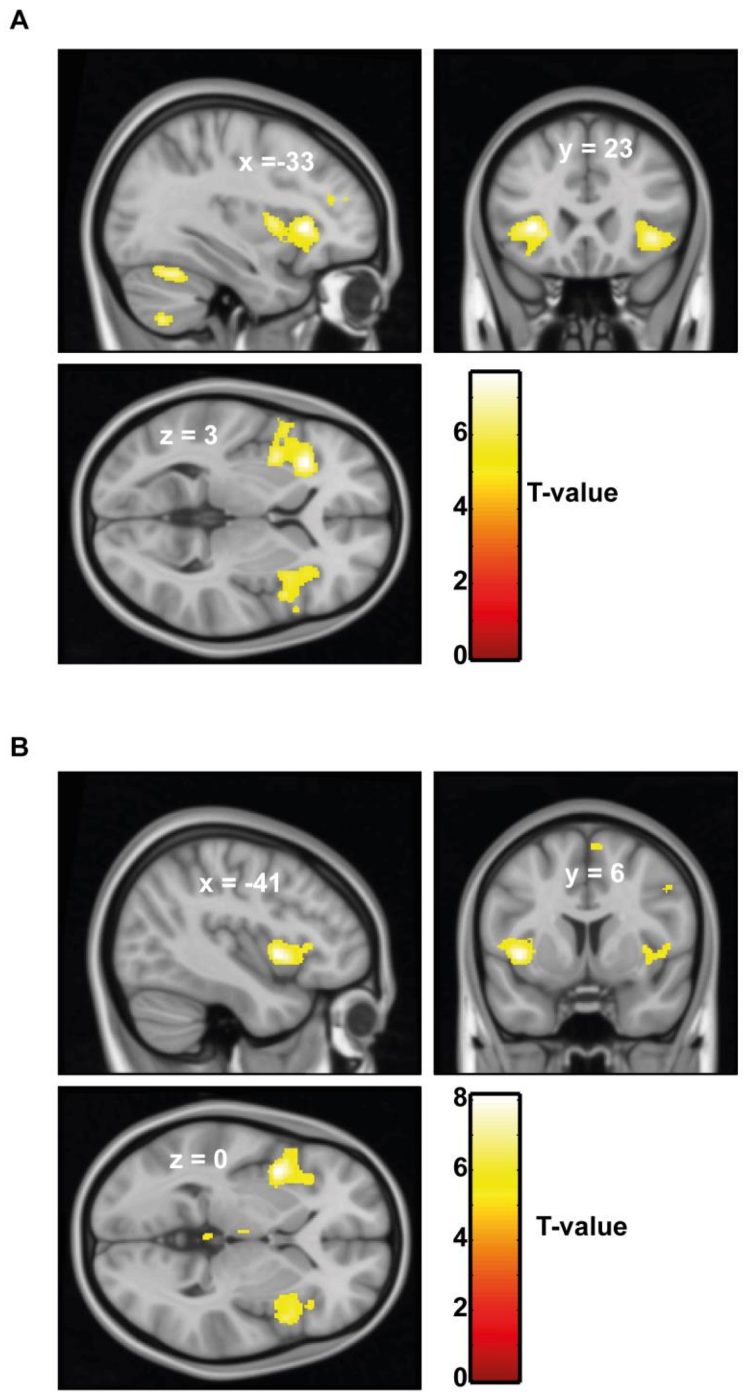

Figure S2: Post-hoc testing for the interaction in GLM analysis; Related to Figure 2. (A) Statistical parametric map showing brain regions with greater BOLD activation in misophonics compared to controls in response to trigger sounds compared to neutral sounds. Specifically the contrast as defined in SPM is: Misophonics (Trigger sounds- Neutral sounds) > Controls (Trigger sounds- Neutral sounds). **(B)** Activation of trigger sounds compared to unpleasant sounds: parametric map for the contrast Misophonics (Trigger sounds- Unpleasant sounds) > Controls (Trigger sounds- Unpleasant sounds). The parametric maps in both panels are thresholded at p =0.05 (FWE corrected for the whole brain volume).

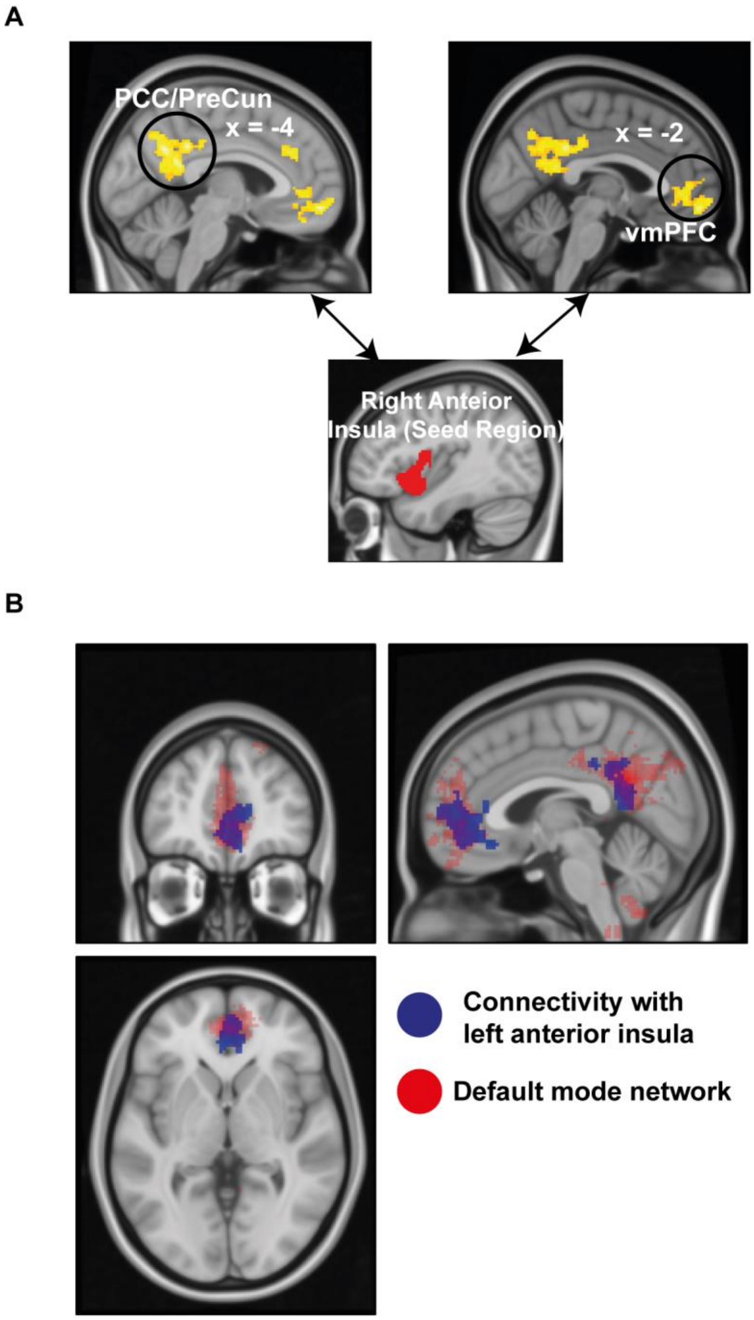

Figure S3: **Functional connectivity of anterior insula and its overlap with default mode network; Related to Figure 3** (A) Functional connectivity with right AIC as a seed region. The figure shows increased connectivity of right AIC to vmPFC and PMC for trigger sounds. The defined contrast is: Misophonics (Trigger sounds- Neutral sounds) > Controls (Trigger sounds- Neutral sounds). The connectivity map is cluster thresholded at $p < 0.05$ with cluster-forming threshold at $p < 0.001$. Other areas which show increased connectivity are, left inferior parietal (-40, 68, 34), anterior cingulate (-6, 34, 28) and parahippocampal cortex (-20, -36, -18). Hippocampus and amygdala are also seen but when the cluster forming threshold is relaxed to 0.005. **(B)** Overlap between the default mode network (DMN) and functional connectivity of left anterior insula shown in Figure 3. The DMN map was obtained using reverse inference from the Neurosynth website (http://neurosynth.org/analyses/terms/default%20mode/).

A B

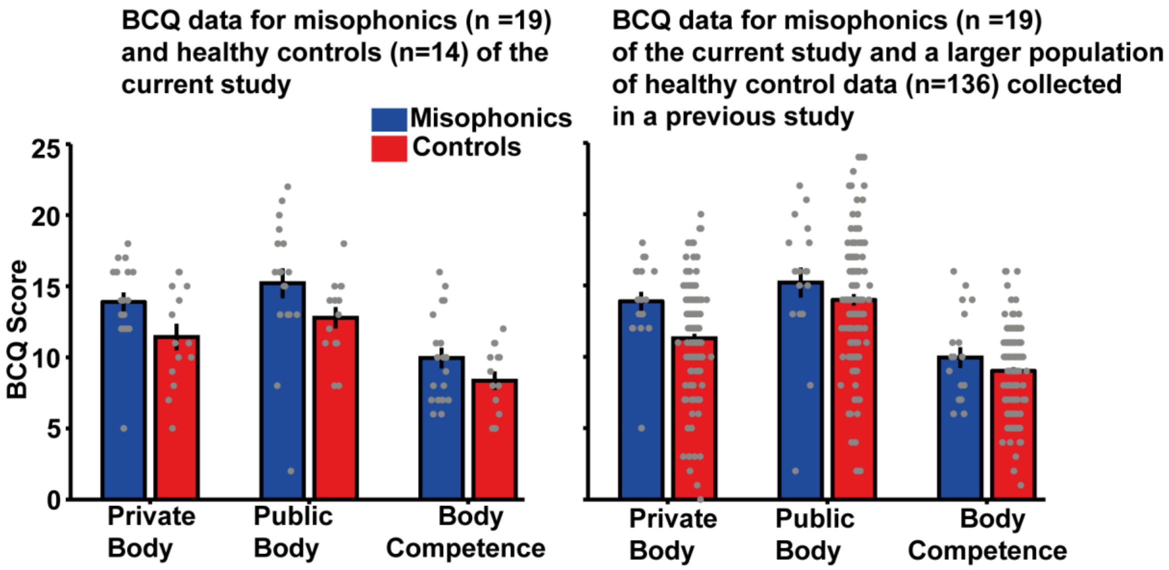

Figure S4: Scores on the three subscales of the body consciousness questionnaire (BCQ); Related to Figure 1. (A) Data from misophonics (n=19) and controls (n=14) who took part in the current study. Statistical comparison (Mann-Whitney U test) between the two groups showed that misophonics scored higher both on the 'private' (p=0.02) and 'public' (p=0.03) categories but were not different from controls on 'body competence' (p= 0.22). **(B)** Comparison of the misophonics scores with data collected from a larger sample (n=136) of normal healthy controls. Misophonics scored higher (p =0.002) on 'private' but were not different from controls in rest of the two categories: 'public' (p = 0.17), 'competence' (p= 0.42). In order to rule out effect of age and gender in the above analysis, we also compared the two groups by tightly matching each misophonic in terms of age (±3 years) and gender of control participants (n=2-132) and looking for systematic effects with the sign test. This comparison showed misophonics scored more highly (p = 0.02) on 'private' but were not different on 'public' (p= 0.65) and 'body competence' (p = 0.36). Dots in the figure show score of individual subjects. For details of the questionnaire, see the section on 'Questionnaire data analysis in supplemental experimental procedure. Data are represented as mean (± SEM).

Table S1: Brain areas which show interaction between group and sound type. The SPM is thresholded at p < 0.05 (FWE corrected for the whole brain volume). Related to Figure 2.

Brain Area name	MNI coordinates	F-value at the maxima	Size of the cluster
Left anterior insula	-41, 6, 0	34.37	1720
Left dorsal anterior insula	-33, 23, 3	30.18	
Left inferior frontal gyrus	-51, 2, 11	27.42	
Left cerebellum	-30, -65, -53	29.20	207
	-27, -65, -24	29.07	522
	-36, -57, -26	26.55	
	-45, -63, -29	16.56	
Right inferior frontal gyrus	48, 11, 2	26.87	1140
Right anterior insula	39, 23, -3	25.18	
Right anterior insula	35, 30,0	19.72	
Right SMA	5, 8, 66	23.90	289
	5, -3, 68	20.35	
Right cerebellum	32, -63, -23	22.47	223
	35, -56, -27	18.91	
	21, -68, -47	19.06	16
	32, -56, -50	18.97	55
	27, -65, -54	16.83	6
Left middle frontal gyrus	-38, 39, 21	20.30	42
Right brain stem	9, -30, -11	19.31	10
	9, -41, -44	16.91	2
	5, -45, -47	16.64	1
Left supramarginal gyrus	-59, -41, 29	18.05	12
Left thalamus	-11, -23, -11	17.49	8
Right precentral gyrus	48, 5, 39	17.19	18
	47, 2, 53	16.68	5
Left cingulate sulcus	-3, 27, 33	16.95	8
Left superior frontal gyrus	-50, 12, 29	16.09	1

Supplemental Experimental Procedures

Subjects

Twenty four misophonic subjects (16 females, mean age 33.8 years, age-range: 18-57 years) and 22 control subjects (15 females, mean age-range 32.5 years, age-range: 19-57 years) participated in the study after providing written informed consent to procedures approved by the local ethics committee. The misophonic subjects were recruited by putting an advertisement on a misophonia support website: http://www.misophonia-uk.org/. Misophonic participants were first required to complete a questionnaire (Please see the supplemental Questionnaire S1). A misophonic participant was recruited for the study if (i) he/she identified sounds of eating, breathing, chewing as trigger sounds (ii) sounds alone could trigger the misophonic reaction (that is, no picture or video of the person producing trigger sounds was needed along with sounds) (iii) the person producing trigger sounds did not have to be a close family member (that is, a stranger producing trigger sounds could produce similar if not the same reaction). The controls were recruited by advertising on a local university website. In the advertisement, the exact purpose of the study was not mentioned. Instead it was stated that the objective of the study was to determine brain responses to our day-to-day environmental sounds. Once participants signed up for study, they were asked how they respond to environmental sounds including sounds of eating, breathing. If typical symptoms of misophonia were absent (e.g. responding angrily, leaving the place) the subject was recruited. No subject who signed up for the study was incidentally diagnosed as misophonic. The misophonics and controls were matched in age and sex. Four misophonic subjects could not be included in the analysis because one subject did not complete the full paradigm (because of emotional distress) and three subjects moved excessively in the MRI scanner. Participants were paid £10/hour plus travel expenses, if any, for their participation.

Stimuli

One objective of the study was to test if misophonic reactions were related to the typical annoyance that is experienced by most people when listening to certain sounds such as sound of a person screaming or a baby cry. Our experiment, therefore included a set of unpleasant sounds (expected to prove annoying to both misophonic and control group) in addition to a set of common misophonic trigger sounds which evoke a misophonic reaction in susceptible individuals. Additionally a set of affectively neutral sounds were used as control sounds. In summary, the experimental stimuli consisted of three sets of sounds (1. Trigger sounds, e.g. eating, breathing, drinking sounds) 2. Unpleasant sounds, e.g. baby cry, a person screaming 3. Neutral sounds, e.g. sounds of a busy café, rain sound), each consisting of 14 stimuli. The trigger sounds were recorded in our lab while people were eating, breathing or chewing. The unpleasant and neutral sounds were downloaded from internet websites. Sounds were sampled at a rate of 44.1 kHz and trimmed to 15s duration.

Experimental Procedure

The study was divided in two parts and required participants to visit our lab on two separate days (24-48 hours separation between the two parts). The reason for dividing the study into two parts was that we were not sure how the misophonic subjects would respond to trigger sounds in the confined space of MRI scanner. In the first visit subjects were acquainted with the sounds and the MRI scanning environment and in the second visit fMRI data were acquired while subjects listened to the three sets of sounds.

In the first visit, the full paradigm was explained and after informed consent was obtained subjects were seated in a sound-proof room. Two Ag/AgCl electrodes were placed on the middle and ring fingers of left hand to monitor galvanic skin response (GSR). All three sets of sounds were played binaurally over headphones (SENNHEISSER, HD380 Pro http://en-uk.sennheiser.com/headphones) in a pseudo-randomized order using the following paradigm. The start of a trial was indicated by text instructions appearing on the screen (‘Sound to Start Soon’). After a gap of 5-7s (chosen randomly), sound was played for 15s. After sound offset, subjects were prompted to give two sequential ratings on a scale from 1 to 4 with a right-handed button press. In the misophonia group the two ratings were (i) ‘how annoying the sound was’ (“1: Not annoying” and “4: highly annoying”) (ii) ‘how effective the sound was in triggering misophonic reaction (“1: Not at all” and “4: Highly effective”). The

procedure for control subjects was same except for the second rating when they rated the 'ant-socialness' of the sound in the sense that they would not like to be in the environment in which this sound is made. This task was chosen as it made no sense to ask the non-misophonic participants about misophonic experiences and misophonic subjects have a strong tendency to escape from the environment in which trigger sounds are made. A total of 4 sessions (126 trials) each lasting ~12 minutes were used. After this session, subjects were taken to MRI scanner and a structural scan using Multiparameter maps (MPM) [S1] was acquired which took about ~25 minutes. The GSR data collected in this session is not presented here.

In the second visit, after attaching two Ag/AgCl electrodes on the left middle and ring fingers for monitoring GSR and an MR-compatible pulse oximeter (Nonin Medical; Minnesota, USA) on the distal left index finger, subjects lay in the MRI scanner and EPI data were acquired continuously while all three sets of sounds were played binaurally in a pseudorandom order through MRI compatible headphones (http://www.mr-confon.de/en/products.html) at a comfortable volume of approximately 75dBA. As in the first session outside the scanner, after each sound offset subjects were prompted to give two ratings using an MRI-compatible 4-button response pad. The total scanning time was ~56 minutes (divided into 5 sessions).

Functional imaging data acquisition

All imaging data were collected on Siemens 3 Tesla Tim whole-body MRI scanner (Siemens Healthcare, Erlangen Germany) at the Wellcome Trust Centre for Neuroimaging. Functional MRI data were acquired continuously with a 12 channel coil using a sequence that was optimized for acquisition from amygdala and orbitofrontal cortex [S2]. Subject movement was discouraged by instruction and by use of soft padding around the head within the headcoil. The acquisition parameters used were (TR=3.36s; in-plane resolution=3mm isotropic; TE=30ms; 48 slices (covering the whole brain); matrix size=64x74; echo spacing=0.5ms; orientation=transverse; slice tilt=-30° relative to the AC-PC line). A total of 1005 volumes were acquired across 5 sessions. Fieldmaps were acquired (parameters: short TE=10ms; Long TE=12.46ms; polarity of phase-encoding blips=-1; EPI readout time=37ms) for every subject after third session.

Structural data acquisition

A whole brain quantitative MPM protocol (with 32 channel headcoil) was used which consisted of a total of 5 sequences: three FLASH sequences and two calibration sequences for correcting field inhomogeneities [S3,S4]. The three FLASH sequences were respectively proton density (PD), magnetization transfer (MT) and T1 weighted by choosing appropriate values of repetition time (TR) and flip angle (α) for each of them. The repetition times and flip angles for the three FLASH sequences were (PD: TR=23.7ms, α=6°; MT: TR=23.7ms, α=6°; T1: TR=18.7ms, α=20°). For the MT-weighted acquisition, a Gaussian RF pulse of 4ms duration and 220° nominal flip angle was applied 2 kHz off-resonance before non-selective excitation. Gradient echoes were acquired with alternating readout gradient polarity at 6 equidistant times between 2.2ms and 14.7ms. For PD-weighted acquisition, two additional at 17.2ms and 19.7ms were acquired. A high readout bandwidth of 425Hz/pixel was used to reduce off-resonance artefacts. During acquisition subjects were encouraged to be as still as possible with eyes open or closed.

MRI data analysis

Functional imaging data analysis were carried out using SPM12. (http://www.fil.ion.ucl.ac.uk/spm/software/spm12/). After discarding the first three volumes to allow for magnetic saturation effects, the remaining images were realigned and unwarped to the first volume to correct for movement of subjects during scanning. Realigned images were then normalized to stereotactic space corresponding to the Montreal Neurological Institute "ICBM152" with parameters estimated from the structural scans and finally smoothed with a 3D Gaussian kernel of 6mm full-width at half maximum. After pre-processing the general linear model (GLM) was used for statistical analysis. The design matrix comprised events using a boxcar function

convolved with the canonical haemodynamic response function provided in SPM. Motion parameters estimated during the realignment step were included in the design matrix.

In the GLM analysis we modelled each of the three types of sound (trigger, unpleasant and neutral) as a separate event of duration 15s with silent periods as implicit baseline. The design matrix also included button presses and motion regressors as regressors of no-interest. A high pass filter with a cut off frequency of 1/128 Hz was applied to remove low frequency fluctuations in the BOLD signal. Once the GLM for each subject was estimated, whole-brain random effects analysis was implemented by entering contrasts of parameter estimates for each individual subject into second-level *F* and *t-tests.* Interaction between group and sound type was computed using a 2x3 ANOVA with group (Misophonic and Controls) and sound type (trigger, unpleasant and neutral) as factors, and subject effects modelled. Post hoc comparison of activity for simple effects between the two groups was done using two sample t-tests.

Functional connectivity analysis was performed using CONN toolbox [S5] (v 15.d). The time series was extracted from anatomically defined ROI (as given in SPM12, neuromorphometrics toolbox) of anterior insula. The effect of movement on the BOLD signal was reduced by regressing out motion parameters, along with their first order temporal derivative, by running whole brain voxel-wise regression. Additionally, five covariates generated using the aCmpCorr method [S6], which uses principal component analysis (PCA) on the measurements made in the white matter and CSF of each individual subjects segmented white matter and CSF masks, were used. The data were then high pass filtered with a cut-off frequency of 1/125 Hz. First-level functional connectivity for each group and condition was computed using bivariate correlation coefficient between the seed time series and time series from all other voxels in the brain. The 'neutral sounds' condition was taken as a baseline and comparison of connectivity between the two groups at second level was undertaken using two sample t-tests.

Structural data were analysed using voxel-based quantification (VBQ) toolbox [S1] in SPM12. Four quantitative maps (effective proton density, PD^*; longitudinal relaxation rate, R1; magnetization transfer saturation, MT and effective transverse relaxation rate, $R2^*$) were computed for each subject in the two groups. Briefly for MT, PD^* and R1 maps, the set of echoes for respective weightings were averaged to increase the signal to noise ratio and the resulting 3 volumes were subjected to the procedure as outlined in [S1,S7, S8] . To obtain $R2^*$ maps, log signal from the 8-PD weighted images was regressed against echo time. For further details of map creation, please see Callaghan et al (2014) [S9]. For performing voxel-based analysis, the MT maps (from each subject) were segmented into gray and white matter probability maps using the unified segmentation algorithm [S10]. Inter-subject co-registration of these tissue segments was performed using DARTEL, a nonlinear diffeomorphic algorithm as implemented in SPM12. This algorithm iteratively creates an average (across subjects) template and determines the deformations that best align the tissue maps to the average template. In our study average template was created by combining images from the two groups. The quantitative maps were co-registered, they were normalized to MNI space using subject specific deformations using a combined probability weighting and smoothing (Gaussian kernel of 3mm FWHM) procedure [S11]. This approach aims to preserve the quantitative values during the normalisation procedure by minimizing any partial volume effects introduced by the smoothing process. Statistically significant differences in tissues between the two groups were assessed using two sample t-tests implemented in SPM12. Age, sex and total intracranial cranial volume were included as regressors of no-interest. We also did a conventional voxel based morphometry (VBM) analysis on the cohort's MT maps, but this analysis did not reveal any significant differences. VBM analyses are dependent on many different parameters (algorithm, priors, and the contrast of the input) and interpretation of any findings, or lack thereof, is complex. Presumably the difference in MT values between the two groups, which could be identified via our quantitative comparison, was insufficient to greatly alter the segmentation result (since the expected MT value of adjacent white matter will be even higher still, e.g. reported as >1.7 in [S12] underpinning the VBM analysis.

Galvanic skin response and heart rate analysis

Galvanic skin response was recorded by placing two Ag/AgCl electrodes on the middle and ring fingers of non-dominant hand. The electrodes were connected to a custom built constant voltage (2.5 volt) coupler. The output of the voltage coupler was converted into an optical pulse frequency with an offset of 125Hz. The pulse signal was digitally recorded using Micro 1401/Spike2 (Cambridge Design) and then converted back to units of

conductance. Heart rate data was measured using a pulse oximeter connected on the index figure of the non-dominant hand and was digitally recorded using Micro 1401/Spike2. The amplifier encoding heart rate and GSR received TTL pulses from the scanner console identifying the current acquisition slice allowing synchronisation of physiological data to the fMRI data and experimental paradigm.

GSR data for 2 misophonic and 4 control subjects could not be used in the final analysis because of technical problems in recording data. Heart rate data for 2 control subjects could also not be used in the final analysis because of technical issues in data recording.

GSR data were first resampled to a sampling frequency of 10 Hz before filtering with a 2nd order Butterworth bandpass filter with cut-off frequencies [0.0015 2.5] Hz. The data were then epoched for each trial from 5s prior to sound onset of sound until 23s after onset. Data were visually inspected (blinded to trial type) and trials with artefacts were rejected. The trials were then sorted into different conditions (Trigger sounds/Unpleasant sounds/ Neutral sounds). After baseline (5s prior to onset of sound) correction, average evoked GSR across trials was computed. Statistical analysis was performed on the evoked GSR time series using a 2x3 ANOVA, as in the fMRI analysis. Correction for multiple comparison was done using cluster level thresholding implemented in SPM for M/EEG with a family wise error (FWE) threshold of $p < 0.05$ and cluster forming threshold of $p=0.05$. This method of correction finds periods of data where more contiguous data points than would be expected by chance pass the cluster forming threshold based upon estimation of the 1-dimensional smoothness inherent in the time series.

HR data were first converted into a continuous time series by inferring instantaneous heart rate based upon the interbeat interval and using spline interpolation with a supersampled 10Hz timecourse. The data were visually inspected for artefacts and periods with average HR < 45 bpm or > 120 bpm automatically rejected. Data were then epoched into trials, and baseline corrected as in the analysis of GSR data. After computing the evoked HR response, it was subjected to statistical analysis using the same procedure as for GSR data.

Mediation Analysis

We used whole brain single-level mediation analysis. Mediation analysis is a 3-variable path analysis [S13] which tests if the relationship between an input (X) and output variable (Y) is mediated by a third variable (M). It compares two models: a reduced model (equation 1) and a full model (equation 2)

$$Y = cX + e_y \qquad (1)$$

$$M = aX + e_M; \quad Y = bM + c'X + e_Y \qquad (2)$$

Mediation analysis tests if the difference:

$$c - c' = ab$$

is significantly different. If the difference is significant then it means that part (or whole) of the variance of Y which is explained by X alone (equation 1) can be explained by the mediator variable M (equation 2, second half) leading to a reduced value of c' compared to c.

In our study we asked which brain regions would explain higher heart rate and skin conductance in misophonic subjects compared to control subjects. For this analysis, our input variable X was a categorical variable (1 for the misophonic -1 for controls). The output variable Y was the average heart rate or skin conductance value for the subject. The mediator variable was the contrast (trigger sound-neutral sound) for each subject. The significance of mediation (ab) was tested using bias corrected bootstrap testing [S14] with 10000 samples at each voxel and the p-value at each voxel was calculated.

Questionnaire data analysis

To evaluate interoceptive sensibility [S15], the body consciousness questionnaire (BCQ) [S16] was administered after completion of the fMRI component of the study. Participants were requested to fill-in the questionnaire online. Nineteen misophonic and 14 control subjects filled in the questionnaire. The BCQ questionnaire has 15 questions in total with 5 questions in each of the three categories: Private Body Consciousness, Public Body Consciousness and Body Competence. The part of the BCQ related to private body evaluates perception of body as perceived from inside and comprises questions like: *I am sensitive to internal bodily tensions*; *I know immediately when my mouth or throat gets dry*. In public body consciousness, perception of body as visible to others is evaluated and consists of questions like: *when with others, I want my hands to be clean and look nice*; *I am aware of my best and worst facial features*. The body competence part of the BCQ evaluates awareness of the physical condition of body and includes questions like: *For my size, I am pretty strong; I am better coordinated than most people*. Each question has 5 options which are scored from 0 (Extremely uncharacteristic) to 4 (Extremely characteristic) thus making a maximum possible score of 60. Data were analysed by comparing the scores between misophonics and controls on the three categories separately using non-parametric equivalent to the two sample t-test (Mann-Whitney U test for unequal medians). Because of relatively small sample size of the control population who completed the questionnaire and to further check the reliability of our results, we also compared the BCQ scores of misophonic participants with scores of a larger population (n=136; 74 females, age range 25 to 63 years) of healthy controls on the same questionnaire. These data are a subset of data collected from healthy control participants by one of the co-authors (JSW) as a part of a separate study; the larger sample includes a large number of younger participants (n=208) who were excluded from this comparison to ensure average age matching between groups (t_{153}=0.5, p=0.61) although we checked that inclusion of the remaining younger control participants made no difference to the pattern of results). In order to further explore differences between misophonic participants and healthy controls, we also performed a type of case-control analysis, in which the scores for each patient were compared to the mean scores of healthy controls within this larger population matched for gender and within 3 years of age. The sign test was then used to assess whether participants with misophonia scored systematically differently from controls for the subscales of the questionnaire; the pattern of results was the same as for the more traditional analysis against a control population.

Supplemental References:

S1. Weiskopf, N., Suckling, J., Williams, G., Correia, M. M., Inkster, B. (2013). Quantitative multi-parameter mapping of R1 , PD* , MT , and R2* at 3T : a multi-center validation. Front. Neurosci. 7, *95*, 1-11.

S2. Weiskopf, N., Hutton, C., Josephs, O., Deichmann, R. (2006). Optimal EPI parameters for reduction of susceptibility-induced BOLD sensitivity losses: a whole-brain analysis at 3 T and 1.5 T. Neuroimage. *33*, 493–504.

S3. Lutti, A., Hutton, C., Finsterbusch, J., Helms, G., Weiskopf, N. (2010). Optimization and validation of methods for mapping of the radiofrequency transmit field at 3T. Magn. Reson. Med. *64*, 229–238.

S4. Lutti, A., Stadler, J., Josephs, O., Windischberger, C., Speck, O., Bernarding, J., Hutton, C., Weiskopf, N. (2012). Robust and fast whole brain mapping of the RF transmit field B1 at 7T. PLoS One. *7*, e32379.

S5. Whitfield-Gabrieli, S., Nieto-Castanon, A. (2012). Conn: a functional connectivity toolbox for correlated and anticorrelated brain networks. Brain. Connect. *2*, 125–141.

S6. Behzadi, Y., Restom, K., Liau, J., Liu, T. T. (2007). A component based noise correction method (CompCor) for BOLD and perfusion based fMRI. Neuroimage. *37*, 90–101.

S7. Helms, G., Dathe, H., Dechent, P. (2008). Quantitative FLASH MRI at 3T using a rational approximation of the Ernst equation. Magn. Reson. Med. *59*, 667–672.

S8. Helms, G., Dathe, H., Kallenberg, K., Dechent, P. (2008). High-resolution maps of magnetization transfer with inherent correction for RF inhomogeneity and T1 relaxation obtained from 3D FLASH MRI. Magn. Reson. Med. *60*, 1396–1407.

S9. Callaghan, M. F., Freund, P., Draganski, B., Anderson, E., Cappelletti, M., Chowdhury, R., Diedrichsen, J., Fitzgerald, T. H., Smittnear, P., Helms, G. et. al. (2014). Widespread age-related differences in the human brain microstructure revealed by quantitative magnetic resonance imaging. Neurobiol. Aging. *35*,

1862–1872.

S10. Ashburner, J., Friston, K. J. (2005). Unified segmentation. Neuroimage. *26,* 839–851.

S11. Draganski, B., Ashburner, J., Hutton, C., Kherif, F., Frackowiak, R. S. J., Helms, G., Weiskopf, N. (2011). Regional specificity of MRI contrast parameter changes in normal ageing revealed by voxel-based quantification (VBQ). Neuroimage. *55,* 1423–1434.

S12. Weiskopf, N., Suckling, J., Williams, G., Correia, M. M., Inkster, B., Tait, R., Ooi, C., Bullmore, E. T., Lutti, A. (2013). Quantitative multi-parameter mapping of R1 , PD*, MT, and R2* at 3T : a multi-center validation. Front. Neurosci. *7,* 1–11.

S13. Baron, R. M., Kenny, D. A. (1986). The moderator-mediator variable distinction in social psychological research: Conceptual, strategic and social considerations. J. Pers. Soc. Psychol. *51*, 1173–82.

S14. Efron, B., Tibshirani, R. (1993). An Introduction to the Bootstrap. (New York: Chapman & Hall/CRC Press).

S15. Garfinkel, S. N., Tiley, C., O'Keeffe, S., Harrison, N. A., Seth, A. K., Critchley, H. D. (2015) Discrepancies between dimensions of interoception in Autism: Implications for emotion and anxiety. Biol. Psychol. *114,* 117-126.

S16. Miller, L. C., Murphy, R., Buss, A. H. (1981). Consciousness of body: Private and public. J. Pers. Soc. Psychol. *41*, 397–406.

Supplemental Misophonia Questionaire S1

Name : ______________________________________

Age : ______________________________________

Sex: ______________

Q1: Please list the sounds that you dislike most (the best trigger sound first)

Q2: Does it make a difference who is making these sounds? (e.g. whether a close friend or family member makes them, or a stranger). If 'yes', please explain how it affects your reaction.

Q3: Could you plz rate on a scale, from 1 to 10 (1: no effect; 10 = maximum effect), your reaction when the trigger sound is produced by

i) A stranger __________________

ii) A close family member __________________

Q4: If we have to produce a strong trigger in our lab (in a MRI scanner for example) so that your brain activity under that condition could be monitored, do you think it could be done if

i. **The recorded sounds alone, of the person who triggers strong reaction, are played back to you**
ii. **Just a picture of the person is shown.**
iii. **A silent video of the person doing the action that acts as a trigger (e.g. eating, breathing) is played.**
iv. **Both sounds and picture of the person are used.**
v. **Video with sounds is used**

Please rate each of the above option on a scale from 1 to 10 (1 = no effect; 10= maximum effect)

Q5: Does the situation you are in make a difference to your reaction to these sounds? (e.g. whether you are at work, enjoying leisure activities or trying to relax). If 'yes', please explain how the situation affects your reaction.

Q6: Does the noise level around you affect your reaction to these sounds? (i.e. do you have more or less of a reaction in noisy surroundings).

Q7: If you can, please explain what it is about these sounds that you dislike.

Q8: Please describe the feeling you get when you hear these particular sounds.

Q9: Please describe what you do when you hear these sounds.

Q10: Please describe any steps you have taken to avoid hearing these sounds.

Q11: Please describe the effect that having misophonia has had on your life (including effects on employment, study, hobbies, social activities and relationship with friends and family).

Q12: When did you first notice these symptoms of strongly disliking certain sounds? Was there any particular event or trigger associated with the symptoms starting?

Q13: Have your symptoms changed over time since they began? If so, please explain in what way they have changed (e.g. getting worse, getting better, the reaction itself changing).

Q14: Please list any sounds that you particularly like.

Q15: Do you often experience tinnitus (ringing in the ears)? If so, how much of the time do you hear it, how loud is it (on a scale of 0-10) and how much does it bother you (on a scale of 1-10)?

Q16: As well as particularly disliking particular sounds, do you have a strong dislike of loud sounds in general (ones that other people around you do not seem to mind)?

Q17: Does anybody in your family have similar symptoms to yours? If so, please give details.

Q18: Do you have hearing loss? Have you had a hearing test? If so, what was the result?

Q19: Would you be happy if we contacted you in future to discuss taking part in research on misophonia which requires brain imaging (fMRI or MEG)? If so, please state your preferred contact details (e.g. e-mail address, phone number, postal address).

Q20 . Please provide any additional information.
